# Predicting Long-Time-Scale Kinetics under Variable Experimental Conditions with Kinetica.jl

**DOI:** 10.1021/acs.jctc.4c00333

**Published:** 2024-06-03

**Authors:** Joe Gilkes, Mark T. Storr, Reinhard J. Maurer, Scott Habershon

**Affiliations:** † Department of Chemistry, University of Warwick, Gibbet Hill Road, CV4 7AL Coventry, U.K.; ‡ EPSRC HetSys Centre for Doctoral Training, University of Warwick, Gibbet Hill Rd, CV4 7AL Coventry, U.K.; § AWE Aldermaston, Reading, Berkshire RG7 4PR, U.K.; ∥ Department of Physics, University of Warwick, Gibbet Hill Road, CV4 7AL Coventry, U.K.

## Abstract

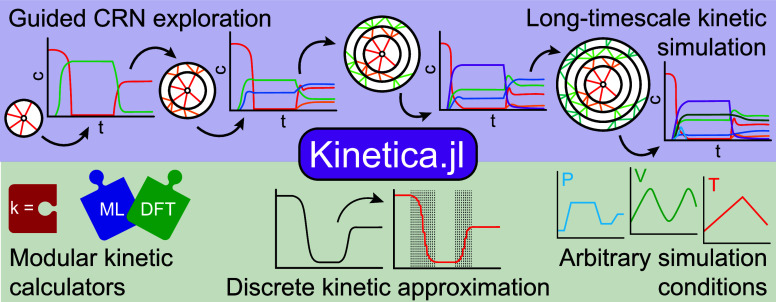

Predicting the degradation processes of molecules over long time scales is a key aspect of industrial materials design. However, it is made computationally challenging by the need to construct large networks of chemical reactions that are relevant to the experimental conditions that kinetic models must mirror, with every reaction requiring accurate kinetic data. Here, we showcase *Kinetica.jl*, a new software package for constructing large-scale chemical reaction networks in a fully automated fashion by exploring chemical reaction space with a kinetics-driven algorithm; coupled to efficient machine-learning models of activation energies for sampled elementary reactions, we show how this approach readily enables generation and kinetic characterization of networks containing ∼10^3^ chemical species and ≃10^4^–10^5^ reactions. Symbolic-numeric modeling of the generated reaction networks is used to allow for flexible, efficient computation of kinetic profiles under experimentally realizable conditions such as continuously variable temperature regimes, enabling direct connection between bottom-up reaction networks and experimental observations. Highly efficient propagation of long-time-scale kinetic profiles is required for automated reaction network refinement and is enabled here by a new discrete kinetic approximation. The resulting *Kinetica.jl* simulation package therefore enables automated generation, characterization, and long-time-scale modeling of complex chemical reaction systems. We demonstrate this for hydrocarbon pyrolysis simulated over time scales of seconds, using transient temperature profiles representing those of tubular flow reactor experiments.

## 1 Introduction

Many industrial components and materials are subjected to extreme environmental stresses from a number of sources, limiting their lifetimes. Heat generated through friction, oxidative conditions, and exposure to different wavelengths of electromagnetic radiation can contribute to permanent material degradation at the molecular level, increasing the potential for critical material failure.^1−7^ While these effects can be studied under controlled experimental conditions, this can involve many long-time-scale trials. Even with such experimental results, it can be difficult to elucidate the microscopic mechanisms that lead to macroscopic degradation phenomena.^8^

Alternatively, degradation processes can be modeled computationally. However, a deep understanding of all potential chemical reactions that a material could undergo during experiments is required to fully capture the emergent chemical kinetics over long time scales. Not only does this understanding demand an expansive chemical reaction network (CRN) of elementary reactions, but it also requires the rates at which these events occur (which are inherently dependent on the experimental conditions, such as temperature, at a given time).^9^

This problem has been the focus of significant research for decades, and many approaches for automated reaction discovery (ARD) have emerged for enumerating all possible reactions from a set of given chemical species.^10^ These can range from exhaustive searches through vast molecular graphs (sometimes referred to as connectivity or adjacency matrices) to molecular dynamics-based sampling techniques.^11,12^ Most modern approaches include a way to selectively explore chemical reaction space, only following reaction pathways that yield kinetically viable products under a given set of experimental conditions. This was pioneered by Susnow et al. in the NetGen code, where estimated rates of formation of each unreacted species are iteratively used to expand a “core” of species that can undergo reaction, growing the CRN only where reactions are predicted to be viable based on approximate rates of species formation.^13^ This approach has the benefit of creating streamlined CRNs that are more easily analyzed and simulated. It has seen use in popular contemporary codes such as the Reaction Mechanism Generator (RMG) and Software for Chemical Interaction Networks.^11,14^

The determination of accurate reaction rates for the reactions sampled by ARD schemes is another point of difficulty when constructing large CRNs.^10^ In the standard approach, reaction rate evaluation requires identification of the transition state for every reaction in a CRN, typically starting from minimum energy path (MEP) schemes such as the nudged elastic band method or the growing string method, which is coupled to a computationally expensive, but accurate, electronic structure method such as density functional theory (DFT).^15,16^ There have been many recent developments that allow for the calculation or prediction of the rate constants within a CRN at reduced computational cost, but we will reserve this discussion for a forthcoming paper in which we investigate the applicability of using machine learning (ML) to predict reaction rates.

A further important challenge associated with simulations of degradation processes comes from the kinetic modeling of CRNs. This is the process of converting a CRN into a time-dependent form and propagating the degradation process in time under a set of given thermodynamic conditions. These forms can include discrete descriptions of every molecule being simulated, such as the chemical master equation (CME) and the stochastic simulation algorithms used to explicitly model every reactive event within a network.^17^ They can also include continuous, approximate descriptions such as the reaction rate equation (RRE), which is solved as a system of ordinary differential equations (ODEs).^18^ Kinetic modeling of CRNs provides invaluable data, such as the distribution of all species in a reaction mixture at any given time. These distributions, as well as statistics like concentration fluxes, can be used to identify potentially hazardous byproducts of chemical degradation processes and avoid their formation.^19^

When modeling degradation over long time scales, however, there is an additional consideration. When simulating the kinetic evolution of a CRN under constant experimental conditions (temperature, pressure, etc.), assuming reaction rates respect the principle of detailed balance, the concentration of every species within the CRN will tend toward a steady state.^20^ Real-world systems of interest are rarely held at constant conditions over extended periods. As such, a complete kinetic analysis of CRNs requires variable experimental conditions to tackle long-time-scale kinetics—that is, kinetics under real-world time scales that exist many orders of magnitude above those at which individual chemical reactions are taking place. These variable conditions should be implemented to allow for arbitrary state variables to be utilized in kinetic simulations, such that simulations can be extended to take into account the effects of interesting and realizable changes in experimental conditions.

Kinetic simulations with variable experimental conditions are not often a priority of many contemporary CRN exploration codes, which instead focus on the generation and analysis of complex CRNs,^21−26^ sometimes extending to constant condition kinetic simulations.^11^ On the other hand, codes such as Cantera and CHEMKIN exist for the sole purpose of performing complex kinetic and thermodynamic simulations on already explored networks.^27,28^ While these codes excel at performing reactive flow simulations under variable conditions, they are restricted to using a narrow set of ODE solvers that are not guaranteed to be capable of handling the solution of expansive, extremely stiff RREs over time scales many times greater than those of the individual reactions occurring within them. To tackle these problems reliably, we require access to a large library of ODE solvers that can be interchanged depending on the requirements of the kinetic simulation at hand.

**Figure 1:**
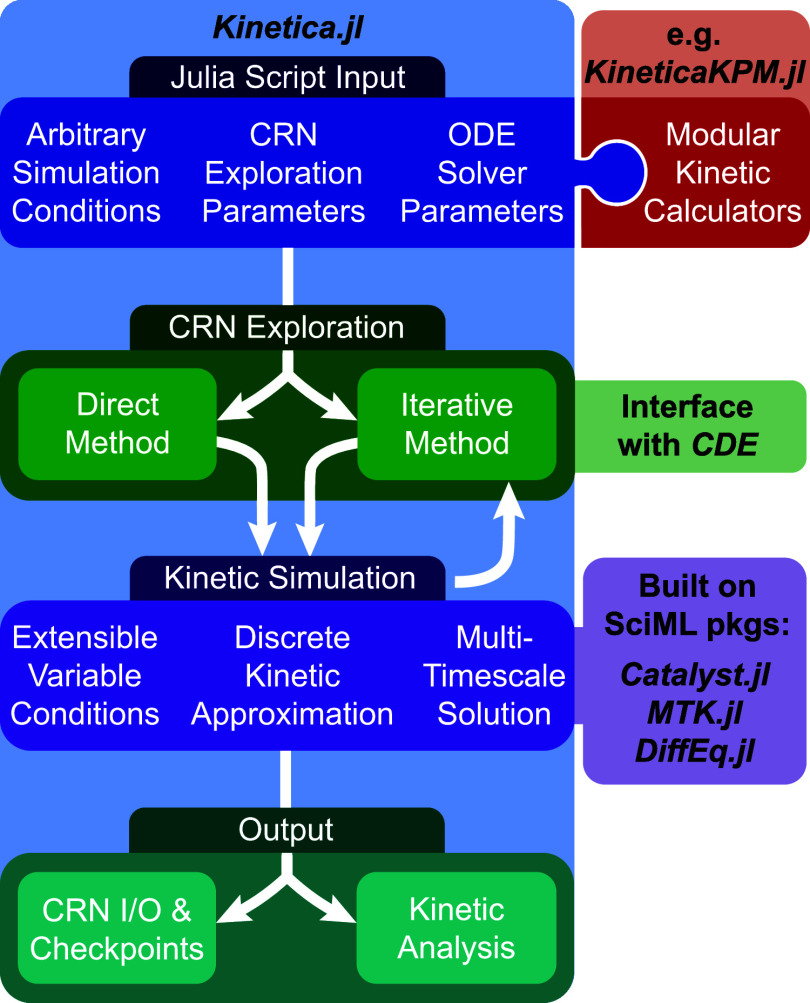
Schematic of the workflow within *Kinetica.jl*, including a breakdown of major features and dependencies. Two dependencies, *ModelingToolkit.jl* and *DifferentialEquations.jl*, are abbreviated as *MTK.jl* and *DiffEq.jl*, respectively.

There is currently no unified code with which both CRNs for arbitrary variable experimental conditions can be automatically generated and long-time-scale kinetic modeling under these conditions can be performed. Here, we present *Kinetica.jl*, a new package written in the Julia language (Figure 1). *Kinetica* combines a kinetics-based reaction exploration algorithm with a molecular graph-driven sampling (GDS) approach to automatically explore the chemical reaction space that is relevant to arbitrary user-supplied variable experimental conditions. It makes use of an existing rich package ecosystem for symbolic-numeric computation and high-performance ODE solving to perform kinetic modeling of large CRNs for the long-time-scale breakdown of molecules with continuously variable conditions. The Julia language and its SciML organization are vital to this approach, with packages such as *DifferentialEquations.jl*, *ModelingToolkit.jl*, and *Catalyst.jl* enabling fast simulations with simple low-level access to a plethora of underlying ODE solvers.^29−32^ This flexibility allows us to extend the RRE solution with continuously variable conditions with an approximation that enables fast construction and simulation of large CRNs with negligible loss in accuracy.

This paper details the implementation of many of *Kinetica*’s underlying systems and benchmarks some of the capabilities of the code on a generated CRN for the pyrolysis of ethane. However, *Kinetica.jl* also contains a modular interface for kinetic calculators, separate packages which can be used to extend the available ways in which reaction kinetics can be modeled. We are releasing *Kinetica.jl* alongside the *KineticaKPM.jl* calculator for ML-based kinetic prediction, which uses a previously published activation energy prediction model to enable fast, computationally inexpensive CRN construction.^33^

The remainder of this article is organized to explain each of the major workflow sections shown in Figure 1. In Section 2, we describe two implementations of CRN exploration with our GDS approach, particularly focusing on the coupling between kinetic modeling and reaction exploration to provide an efficient scheme for automated CRN construction. In Section 3, we describe how *Kinetica* performs long-time-scale kinetic simulations of generated CRNs under conditions of variable thermodynamic parameters, offering a route toward experimentally tailored CRNs. Finally, in Section 4, we showcase the end-to-end *Kinetica* simulation workflow for ethane pyrolysis experiments. Finally, we offer some future directions for the further development of *Kinetica*.

## 2 Reaction Mechanism Exploration

*Kinetica* selectively explores chemical reaction space for molecular reactive processes under complex experimental conditions using a two-step approach. First, individual reactions and mechanisms are generated using a single-ended GDS approach, as developed in a previous work.^34^ Second, the generated reactions are aggregated into reaction networks that are selectively explored using an iterative kinetics-based algorithm, delivering extensive coverage of chemical reaction space while maintaining CRN compactness. In the following, the generation of CRNs in *Kinetica* is discussed in the context of molecular degradation processes, but we emphasize that our overall approach is equally applicable to systems of multiple reactive species.

### 2.1 Generating Reactions and Mechanisms

The GDS approach, referred to as the graph-based reaction-path sampling approach in refs (34–38), was originally formulated for use in double-ended reaction path searches. Here, reactant and product Cartesian coordinates (**r**_0_ and **r**_P_, respectively) are transformed into connectivity matrices (CMs, also referred to as graphs **G**^0^ and **G**^P^, respectively). The original double-ended GDS approach (DE-GDS) searches for directed paths through the chemical reaction space that connect the two end-point graphs; each such path represents a candidate reaction mechanism that may be postanalyzed using electronic structure calculations to evaluate and rank mechanistic hypotheses.

In the context of GDS, the connectivity matrices **G** are *N* × *N* square matrices, where *N* is the number of atoms in the system, with elements
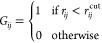
1Here, *r*_*ij*_ is the distance between atoms *i* and *j* and *r*_*ij*_^cut^ is a cutoff distance for atoms of the same types as *i* and *j*, below which the atoms are considered to be bonded (noting that the type of chemical bond is not relevant in this definition). The definition of *r*_*ij*_^cut^ follows our previous work and is given by
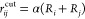
2where *R*_*i*_ and *R*_*j*_ are, respectively, typical atomic covalent radii and α = 1.1 is a parameter that enables some flexibility to account for the observation of different bond lengths in different molecular environments. This simple approach to defining bonding cutoff distances has been used extensively in our previous work on graph-driven reaction network exploration.^34−38^

Using a simulated annealing procedure, a sequence of CMs can be stochastically modified to generate potential mechanisms comprising elementary reactions such that reactant and product CMs, **G**^0^ and **G**^P^, are ensured to be connected. By geometry optimization of the reaction-path intermediates, for example, using semiempirical electronic structure methods, this approach delivers sequences of reactive intermediates, connected by elementary reactions, that form a low-energy path through chemical reaction space. Reaction paths generated in this way can subsequently be complemented with schemes to identify the MEP between each intermediate structure along the path. The DE-GDS algorithm is implemented in the Chemistry Discovery Engine (CDE) code and has previously been used to investigate, for example, catalysis and reactions in the interstellar medium.^35,36,38^

**Figure 2:**
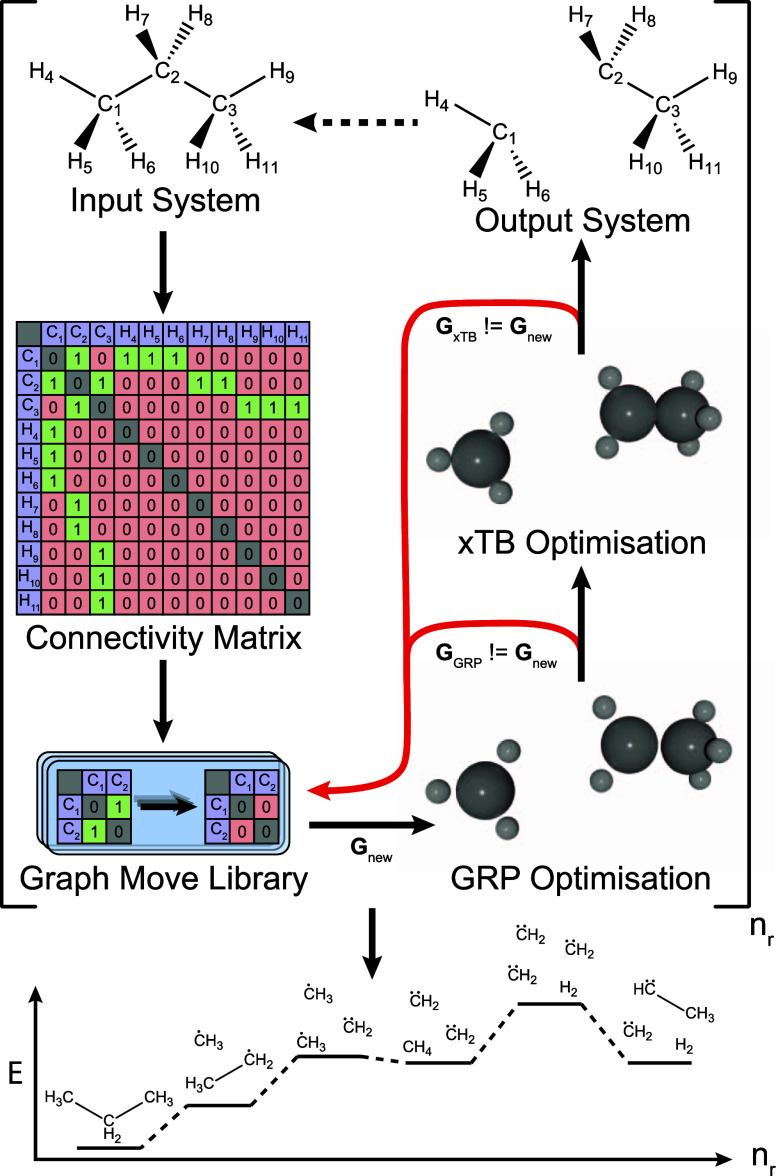
Schematic of the SE-GDS workflow within CDE.^34−38^ A CM is extracted from an input system and a graph move is sampled from the graph move library. The move is applied to generate a new CM **G**_new_, which is converted back into a geometry by successive optimization with the GRP and with GFN2-xTB.^39^ If either changes the CM, a new graph move is sampled. The resulting geometries can be fed back in as inputs *n*_r_ times to generate a random mechanism.

While the DE-GDS method, by definition, yields reactions and intermediates that connect known reactants and products, the automated high-throughput generation of arbitrary CRNs instead demands a method that is suitable for generating reactions when there is no known end-point. We have therefore extended CDE with a single-ended GDS (SE-GDS) algorithm, the aim of which is to generate a CRN given only input knowledge of reactants. This uses many of the same principles as the double-ended approach but can be used without requiring knowledge of a final target structure. The SE-GDS algorithm is outlined in Figure 2.

The SE-GDS algorithm begins by taking an input molecular geometry and calculating its CM. We then select a “graph move” to perform on this matrix. A graph move is defined as a specified change in the CM to a new state. Graph moves can be simple, such as making or breaking a bond between a randomly selected pair of atoms, or can be more complex, such as four-atom reactions typified by the dissociation of molecular hydrogen from across a carbon double-bond. Following our DE-GDS approach, mechanistic exploration uses a library of graph moves representing the allowed chemical reactivity; these moves can be tailored to explore particular types of chemical reaction or can be simply left to be as broad as possible to promote wider exploration of novel chemical reaction paths. Furthermore, as well as supporting the specification of any number of allowed graph moves, our approach also allows users to define the elements that each graph move applies to, offering further routes to tailoring reaction exploration across the range from focused mechanistic hypothesis exploration to large-scale CRN generation. While the graph move library was used for stochastic selection of CM modifications in previous DE-GDS simulations, we have modified it here to also account for a relative probability of each graph move being selected during mechanism generation. This allows us to bias the selection of graph moves toward reaction types that are expected to be more or less prevalent in a given molecular system (again, noting that this bias can also be removed to drive unbiased CRN exploration).

Once a graph move (Figure 2) has been randomly selected, a corresponding set of atoms is chosen to participate in the selected reaction. The graph move is then applied to the current CM of the system, and the resulting CM is verified to ensure that no modified atoms violate any user-defined valence or bonding constraints; for example, a typical constraint is to ensure that carbon atoms are bonded to no more than four other atoms, thereby respecting standard atomic valencies. The resulting CM is also checked against an (optional) list of forbidden graphs (or bonding patterns), which can be used to stop the formation of any unwanted or nonphysical bonding patterns.

If these checks are passed, an approximate molecular geometry for the system after application of the reactive move is generated using a graph restraining potential (GRP); this is a simple empirical potential that is designed to be minimized if a particular set of atomic Cartesian coordinates **r** correspond to a target CM, **G**. In other words, minimization of the GRP for target graph **G** with respect to the atomic coordinates **r** results in a structure for which the calculated CM matches **G**. The GRP we use here is similar to that deployed in our previous studies,^34^ and takes the following form
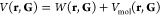
3Here, *W*(**r**,**G**) is an atomic constraining potential that provides a force to move atomic coordinates **r** to match the connectivity in **G** and *V*_mol_(**r**,**G**) is a molecular constraining potential, which ensures that individual molecules are kept sufficiently far apart such that no bonds can form between their constituent atoms.

The GRP allows us to quickly create approximate geometries of newly sampled molecular products from their CMs only. Once new structures have been successfully generated by optimization under the GRP, the resulting (typically high-energy) geometries can then be optimized to stable geometries using an electronic structure method. If any of the valence-based validity checks fail after GRP and electronic structure-based optimization, or if the CM proposed by the graph move is modified by either of these optimizations, the CM is restored to its original form (before the new reaction was imposed) and a new graph move is generated until either a valid graph move is identified or a maximum number of attempts have been made (suggesting that the graph move library cannot be successfully applied to any subgraph of the current CM).

This process of CM generation, graph move selection/validation, and geometry optimization forms one iteration of the single-ended mechanism search, which can be repeated for a user-specified number of iterations *n*_r_ to build up a randomly sampled mechanism of reactions with optimized intermediates from a given starting system. Finally, this whole workflow can be repeated *n*_m_ times to generate multiple mechanisms (each comprising *n*_r_ reactions).

The importance of geometry relaxation of GDS intermediates cannot be overstated as it effectively sanitizes GRP predictions. Because the GRP is biased to generate a geometry that respects the new CM for the system, it can occasionally lead to unstable molecular geometries that isomerize when optimized on a more accurate potential energy surface (PES). For this reason, we employ the semiempirical GFN2-xTB method^39^ to perform the secondary optimizations within the SE-GDS as it has been shown that it often produces atomic forces and optimized geometries similar to DFT within small-to-medium-sized molecules while also exhibiting excellent computational efficiency. Given that a single run of the SE-GDS algorithm deployed here typically requires more than 2*n*_r_*n*_m_ secondary optimizations to verify GRP accuracy, the GFN2-xTB method represents a good balance between accuracy and computational cost. As a representative example, a SE-GDS run with a 15-mer of polyethylene with *n*_r_ = 400 and *n*_m_ = 1 yields a mechanism (i.e., set of GRP- and PES-optimized intermediate structures) in a matter of minutes on a modern consumer-grade computer.

### 2.2 Network Assembly

**Figure 3:**
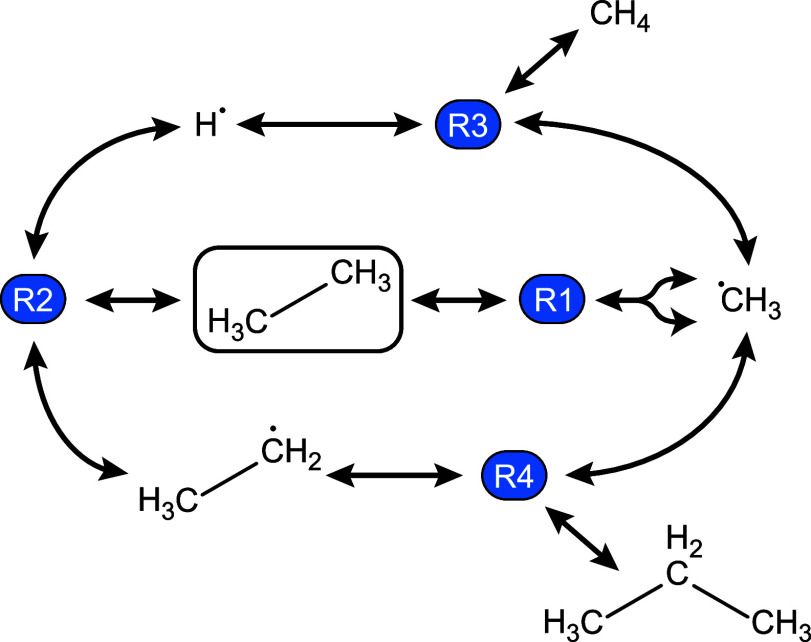
Example of a potentially infinite CRN. Free radical species, formed by homolytic cleavage of bonds in ethane, can combine to form hydrocarbons larger than the original reactant—in R4 an ethyl radical can form a bond with a methyl radical to become propane. This process can be repeated to continue forming larger hydrocarbon chains.

With a method for generating possible reactions in hand, it is possible to think of many ways in which a reaction network could be assembled. However, the stochastic nature of the SE-GDS process means that the way in which chemical reaction space is sampled plays an important role in how complete (or “correct”) a generated CRN is in capturing the reactions important to long-time-scale chemical kinetics. Furthermore, under some simulation conditions, CRNs have the potential to grow indefinitely—unless constraints on system size are imposed—as demonstrated by molecular degradation into free radical species, followed by radical chain growth (Figure 3). Care must therefore be taken to sample chemical reaction space in a way that captures only the chemical reactions that are possible under a set of experimental conditions, rather than seeking to address the combinatorially difficult task of generating *every* possible reaction.

In the next two sections, we discuss two routes to CRN construction using SE-GDS. First, we present a “direct” or brute-force approach; as discussed below, this route quickly runs foul of the challenges associated with the complexity and size of available chemical reaction space. Second, we present an iterative scheme for CRN construction using SE-GDS; this iterative approach is based on using chemical kinetics simulations to grow a CRN along reaction pathways that are the most “kinetically viable”. As we show later, this iterative approach results in focused CRNs that better reflect the essential kinetics of complex variable-condition degradation processes.

#### 2.2.1 Direct Exploration

**Figure 4:**
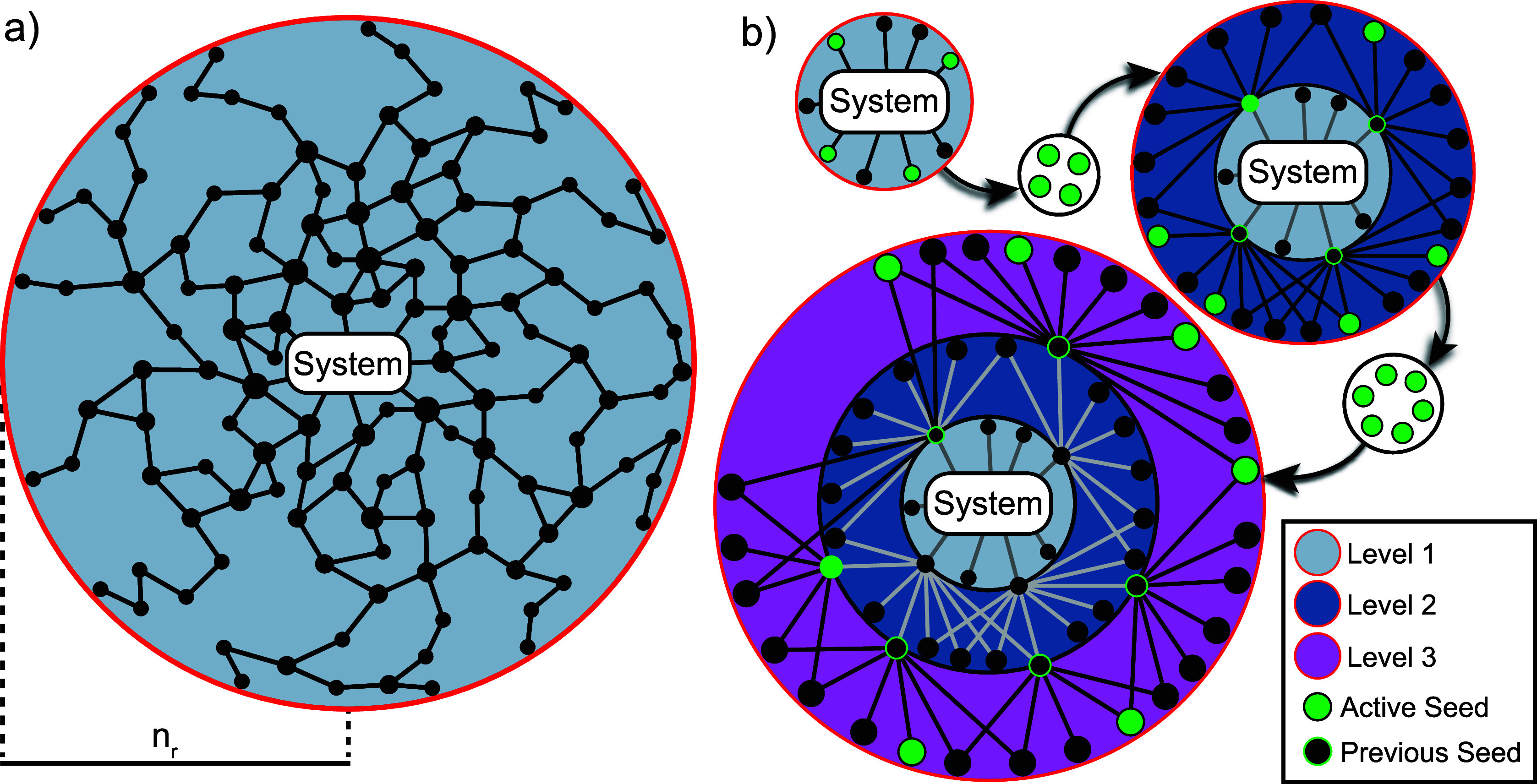
Schematics of the two CRN exploration methods provided in *Kinetica*. (a) The direct method uses SE-GDS to generate random mechanisms of length *n*_r_ from a starting system of molecules until the CRN is converged. Stochastic sampling leads to under-converged species on the outer edge of the explored reaction space. (b) The iterative method proceeds in levels, with each level sampling the reactions adjacent to (*n*_r_ = 1) the high concentration species (seeds) of the previous level and running a kinetic simulation to determine the seeds for the next level, avoiding under-converged species above a threshold.

The simplest method of building a CRN for chemical breakdown reactions is to use SE-GDS to sample as many mechanisms as possible, each for a defined number of reactions *n*_r_ (Figure 4a). Here, *n*_r_ can be thought of as an effective “radius” that defines the outer bounds of explored reaction space. By sequentially adding newly discovered species to the growing network and connecting them with the sampled reactions, vast CRNs can easily be constructed. We typically ignore reactions of a higher molecularity than bimolecular reactions because their probability of occurrence in reaction mixtures is negligible,^40^ although this is easily modified.

This method is perfectly valid for small CRNs where every possible species is only a few reactions away from the starting species (*n*_r_ ≤ 10); in such cases, the probability of repeatedly generating intermediate species—and their possible reactions—is relatively high. In this way, even by randomly sampling new reactions, the network is likely to converge (such that all possible reactions and species allowed by a given set of graph moves will have been found). This convergence can be determined by repeatedly sampling new mechanisms until no new reactions have been found within a user-defined number of attempts.

However, as *n*_r_ is increased to grow larger CRNs, two problems begin to emerge in this direct method. First, CRNs explored this way should sample every possible reaction between every pair of species, leading to a combinatorial growth of reactions as more species are discovered, which can quickly make kinetic simulations of the resulting CRNs computationally intractable. Second, the combinatorial explosion of reactions results in the probability of completely sampling a given species (and all of its reactions) becoming smaller for species that are “further” from the initial reactive species. For example, if a species can only be created by one specific mechanism consisting of many reactions, then completely sampling the reactions of this species relies on being able to repeatedly follow this mechanism in many randomly driven SE-GDS runs. In an ideal scenario with infinite exploration time, all reactions could be found stochastically; with finite exploration time and a user-defined convergence cutoff, the CRN can instead become more sparse as the exploration proceeds away from the initial species. In other words, many species may exist on the outermost discovered “edge” of explored reaction space, and their reactions with both discovered and undiscovered species may be under-sampled.

Under-sampled species are not necessarily a problem if their formation is not kinetically viable—if the concentration of such species in the kinetic simulation of the resulting CRN is always low, then there is no need to sample the further onward reactivity of these species. However, if these under-sampled species are formed in high concentrations during kinetic modeling, they can cause one of two effects. If the reverse reactions leading to decomposition of the under-sampled species have very high rate constants (i.e., they are barrierless, or have very low energetic barriers to reaction), then the species is likely to be so short-lived that any contributions to the kinetics of the CRN from further additional reactions stemming from it are negligible. In this case, the convergence of the CRN would likely not be impacted by the under-sampled species. If, however, there are high activation barriers to these reverse reactions of the under-sampled species (likely if, for example, the formation of the under-sampled species is highly exothermic), then it is likely to have a lifetime within the reaction mixture that supports further reaction. Within the framework of this direct approach to CRN construction, completely sampling these species is only possible by increasing the convergence criteria and waiting for an unlikely chain of reactions leading to this species to be found again, so that further reactions can be discovered for the under-sampled species.

As we show later, these problems are significant enough to dramatically influence the emergent kinetics for CRNs sampled using the direct method in combination with SE-GDS. As such, in the following, we propose an alternative iterative approach for large-scale CRN construction and simulation.

#### 2.2.2 Iterative Exploration

*Kinetica* implements an iterative exploration algorithm that uses the results of a kinetic simulation at each iteration to identify high-concentration species, the reactions of which are explored and added to the growing CRN in the next iteration. This algorithm (Figure 4b) begins by using SE-GDS to explore a subspace of reactions with species adjacent to the starting species (where adjacent species are those that are a single elementary reaction away, so *n*_r_ = 1), until convergence is deemed to have been reached in the same way as was presented in the direct exploration method. These reactions form the initial level of the overall CRN. This level is then kinetically modeled at the experimental conditions of interest, and all species with maximum concentration over the entire simulation (*c*_max_) greater than a user-defined concentration cutoff (*c*_select_) are marked as “seed” species. These seed species are collated into a single seed system, which is then passed back to SE-GDS as starting structures for the next level of CRN exploration.

Within subsequent levels of exploration, reactions adjacent to the input seed system are sampled until level convergence, and a new kinetic simulation is performed using the combined CRN created by adding the reactions and species explored in the current level to those from previously explored levels. The seed species for the next iteration are again identified and collated into a new seed system. Seeds can be selected from anywhere in the currently explored CRN, meaning it is common to see seeds persisting throughout multiple levels of exploration when reactions have not been found that significantly reduce their maximum concentrations. Similarly, if new reactions are discovered that reform a previous seed, such a seed can also re-enter the current seed system and be exposed to reactions with other more recently discovered species.

The concentration cutoff *c*_select_ must be chosen carefully, however, as setting this cutoff too high selects too few seed species at each iteration, leading to an incomplete network. On the other hand, setting the cutoff too low would select too many seed species at each iteration, resulting in excessive numbers of levels being explored (at high computational cost). Only species with final concentrations above *c*_select_ should therefore be considered accurate, as molecules that have not been selected as seeds can be potentially under-sampled. In practice, we find that it is best to run multiple iterative explorations with decreasing values of *c*_select_, such that an acceptable trade-off can be established between CRN complexity and accuracy.

Iterative CRN growth continues until the CRN is converged—that is, all reactions and species relevant to the reactivity of the initial species under a set of experimental conditions have been identified. Unlike the convergence criteria used in the direct method or level convergence, iterative CRN convergence is indicated when no further changes occur across a user-defined number of consecutive seed systems, indicating that the current seeds represent the high-concentration species that will be present in the full CRN. This iterative method has the added benefit of selectively exploring chemical reaction space and only following kinetically viable paths, negating much of the effect of the combinatorial growth seen in the direct exploration method and leading to fewer reactions required in the final CRN, in turn allowing for more efficient kinetic simulations (see Section 3).

#### 2.2.3 Comparison to Contemporary Methods

As noted previously, kinetically driven reaction exploration algorithms like the above have already seen extensive use in popular software codes such as NetGen and RMG.^11,13^ These codes differentiate between “reacted” species (those inside a seed system) and “unreacted” species (those outside of a seed system) by using estimated reaction flux to determine the species that are of kinetic importance. At each iteration, unreacted species with reaction flux above a user-defined cutoff are added to a “core” of reacted species, for which all possible reactions are subsequently generated by matching functional groups within reactants to reaction templates that can be applied in a similar way to the graph moves of SE-GDS to generate product structures.

However, these codes are limited to performing CRN construction under a fixed set of experimental conditions. The CRN exploration algorithms they implement would not be applicable for the long-time-scale simulations under variable conditions that we wish to achieve. Because reacted species are never removed from the reactive core in this algorithm, the number of new species pairs that must be considered at every iteration, *p*_new_, is
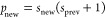
4where *s*_prev_ is the number of species in the previous iteration’s core and *s*_new_ is the number of species being added by the current iteration. Since each species pair can produce many reactions, the sampling process in each iteration becomes progressively more expensive as the core grows. This is less of a problem in static kinetic simulations, especially if the core remains relatively small. However, in variable kinetic simulations, the continuously changing rate constants of reactions could yield high concentration fluxes over the timespan of each kinetic simulation, leading to many of the unreacted species in each iteration being added to the core. Since both *s*_prev_ and *s*_new_ would therefore be large in each iteration, many new species pairs (each with many new reactions) would have to be considered in the next iteration.

In contrast, we anticipate that our iterative approach, using species’ maximum concentrations and refreshing the core (i.e., seed system) at each iteration, will result in a much smaller subset of reactions added in each iteration. This additional granularity is vital for achieving efficient CRNs that can be kinetically modeled over long time scales under challenging experimental conditions, as we demonstrate below.

## 3 Kinetic Modeling

To perform kinetic modeling of a CRN, generated by the SE-GDS-based approaches described in Section 2, it must be transformed into a form that can be numerically integrated with time. When considering simulations of large CRNs in the continuous concentration domain, this typically involves transforming a network into a reaction rate equation (RRE), which acts as a deterministic approximation of the underlying stochastic CME.^17^ In the CME, each reaction is stochastically sampled and resolved sequentially by directly updating the number of molecules of the affected species in the reaction mixture. In the RRE, all reactions are considered to be occurring simultaneously and each species has a continuous concentration gradient which dictates how its concentration evolves at future points in time.

RREs are typically formulated as a set of coupled ODEs. Consider the following toy CRN
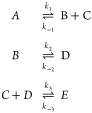
where *A*, *B*, *C*, *D*, and *E* represent five chemical species, and *k*_1_, *k*_2_, *k*_3_, *k*_–1_, *k*_–2_, and *k*_–3_ represent the mass action rate constants^41^ for three forward and backward reactions, respectively. The resulting RRE for this system would be
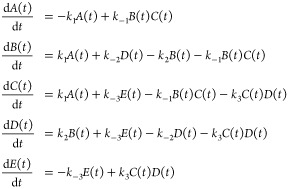
5

The set of RREs above are trivial to solve with many modern computational ODE solvers. However, as the number of species in a CRN increases, the number of reactions connecting them is expected to increase dramatically. This results in many more terms per ODE in the resulting system. Additionally, rate constants in large CRNs often span many orders of magnitude, yielding complex and stiff systems of ODEs that are nontrivial to propagate.

*Kinetica.jl* makes heavy use of packages in the Julia language’s SciML organization to perform these kinetic simulations.^30^ We begin by composing sampled reactions together using *Catalyst.jl*, a symbolic modeling package which allows us to programatically construct and analyze arbitrarily sized CRNs.^32^ The resulting ReactionSystem objects are passed to *ModelingToolkit.jl*, a package for performing high-performance symbolic-numeric computation.^31^ Within *ModelingToolkit.jl*, we can compile a ReactionSystem into a symbolic ODESystem, where the time-dependent species concentrations are represented by symbolic variables (runtime-determined continuous values), and the rate constants that control their evolution are represented by symbolic parameters (runtime-defined static values). The resulting functions are capable of high-performance computation, and the symbolic nature of this compilation allows the same basic system of ODEs to be used for many calculations with varying parametric rate constants.

Alongside the direct compilation of the symbolic RRE, *ModelingToolkit.jl* can be used to perform a variety of performance optimizations. Within *Kinetica.jl*, *ModelingToolkit.jl* performs automatic sparsity detection such that sparse linear algebra routines can be used to more efficiently integrate the RRE.^42^ Having the full functional form of the RRE also enables *ModelingToolkit.jl* to automatically compile an analytic expression for the Jacobian of the ODESystem. These performance optimizations are vital for performing long-time-scale calculations in an efficient and timely manner.

Once generated, the symbolic RREs are solved by ODE solvers implemented within *DifferentialEquations.jl*, an extensive collection of numerical differential equations solvers implemented both natively within Julia, and also within other high-performance languages such as C and Fortran, packaged together under a common interface.^30^ With this library, the selected ODE solver can be changed with a single line of code, enabling the highest degree of flexibility when solving numerically stiff RREs. *DifferentialEquations.jl* allows binding of numeric values to the symbolic parameters created by *ModelingToolkit.jl*, defining initial conditions (the starting concentration of each species) and the simulation timespan.

With this workflow, it is relatively straightforward to convert an explored CRN into high-performance code that can be numerically integrated to reveal concentration–time profiles for all species in the network. However, because each reaction’s rate constant is implemented as a runtime-defined parameter, these values must be provided for the simulation to commence.

### 3.1 Kinetic Calculators

To calculate individual reaction rate constants, *Kinetica.jl* includes several kinetic calculators; these represent a key element of extensibility within *Kinetica* that enables connection to experimental observables. Each calculator defines the experimental conditions (e.g., temperature, pressure, volume, etc.) that it can accept; users can symbolically pass the values of these conditions at simulation time to calculate rate constants on-the-fly. Crucially, the structure of these calculators is simple to implement, and users can define their own calculators that accept custom conditions to run simulations with custom rate expressions.

*Kinetica.jl* provides a base calculator, PrecalculatedArrheniusCalculator, as an example implementation (see Supporting Information Section S1.1 for the example code). This calculator uses the Arrhenius equation to calculate a rate constant for each reaction at the provided temperature
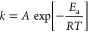
6

It therefore takes a vector of *N* activation energies, a vector of *N* Arrhenius prefactors, and a named argument *T* (representing the temperature of the reaction mixture) and uses them to calculate *N* reaction rate constants, where *N* is the number of reactions in the CRN.

In the case of the iterative CRN exploration algorithm described in Section 2.2.2, the set of reactions—and hence the set of values for *E*_a_ and *A* for every reaction—are not known a priori. It is therefore important to also have calculators that enable determination of rate constants on-the-fly—alongside network exploration. We provide one such calculator in the *KineticaKPM.jl* package, which is capable of quickly estimating reaction rate constants using machine-learned values of *E*_a_ for each reaction, as described in a previous work.^33^ Other calculators could easily be envisioned for performing different roles—for example, a DFT-driven calculator for the evaluation of ab initio rate constants using transition state theory could be easily implemented.

The kinetic calculators described can be called at the start of a simulation, with user-provided fixed conditions, to assign values to the symbolic rate constants in generated RREs; however, this fixes the values of any reaction rate constants for the duration of the kinetic simulation. This simulation technique represents the “standard” kinetic analysis of CRNs but is not applicable for simulating CRNs under variable experimental conditions.

### 3.2 Simulations with Continuous Variable Conditions

To achieve CRN kinetic simulations under variable conditions, *Kinetica* includes a library of parametric condition profiles. These profiles can be flexibly defined as direct functions of time, or as gradient functions that are integrated in time to obtain the final condition profile. All profiles are agnostic to the quantity that they represent and can be bound to a symbolic parameter that allows for their value at any time within a simulation to be passed to the current kinetic calculator. This approach therefore enables kinetic simulations of CRNs to be performed using variable external conditions, providing a clear connection between computation and experiment.

Individual condition profiles are bound together within a ConditionSet. Each ConditionSet can be composed of a combination of condition types.

(a) fixed conditions, where the value of a condition is held constant for the duration of a simulation,
(b) directly variable conditions, where the variation of a condition with time can be expressed analytically,
(c) gradient-variable conditions, where the variation of a condition with time must be integrated from an analytically expressible gradient function.

**Figure 5:**
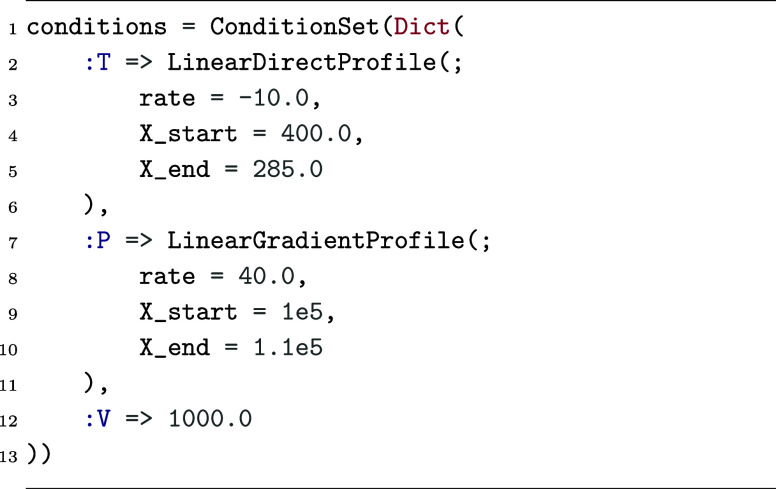
Example ConditionSet definition featuring directly variable temperature, gradient-variable pressure, and constant volume.

These conditions are then passed into one of *Kinetica*’s solvers, where the different condition types can be correctly employed within the RRE propagation. For example, if a simulation was desired in which the simulation temperature decreased, the pressure increased and the volume was held constant, this could be implemented with the ConditionSet shown in Figure 5.

Here, a directly variable temperature profile is used alongside a gradient-variable pressure profile for the sake of demonstration; for simple linear condition profiles, the two can be used interchangeably, but more complex profiles may lend themselves more naturally to a gradient-based definition. The temperature profile generates the following function of simulation time
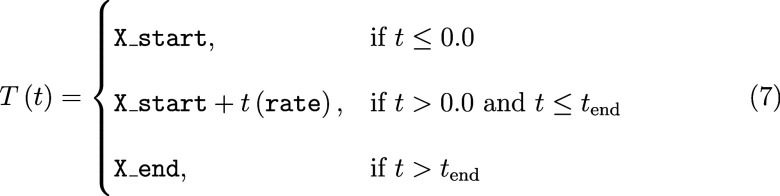
where *t*_end_ = (X_end – X_start)/rate. The pressure profile generates the following function of simulation time
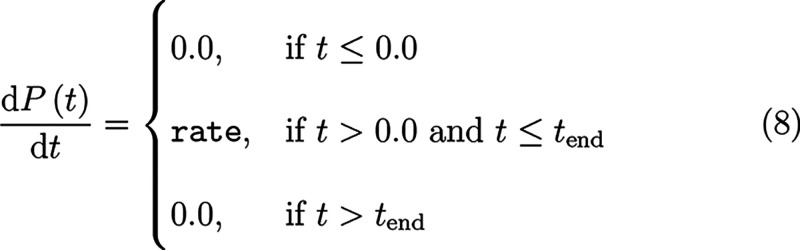
where *t*_end_ is defined the same as above.

#### 3.2.1 Implementation of Variable-Condition Kinetics

We achieve kinetic simulations under variable experimental conditions by incorporating condition profiles like the ones above into the RREs that are generated from CRNs with *Catalyst.jl* and *ModelingToolkit.jl*. This is accomplished within a secondary *ModelingToolkit.jl*ODESystem, where condition profiles are coupled to symbolic variables representing their values (or the values of their gradients, for gradient-based profiles) at a given simulation time. Within this ODESystem, the symbolic rate constants are also bound to the output of the kinetic calculator, when called with the symbolic variable conditions as function arguments. Together, this forms a system of differential algebraic equations (DAEs), which can be integrated in time to calculate the rate constants of the reactions in the current CRN as they vary with the conditions provided in the input ConditionSet.

**Figure 6:**
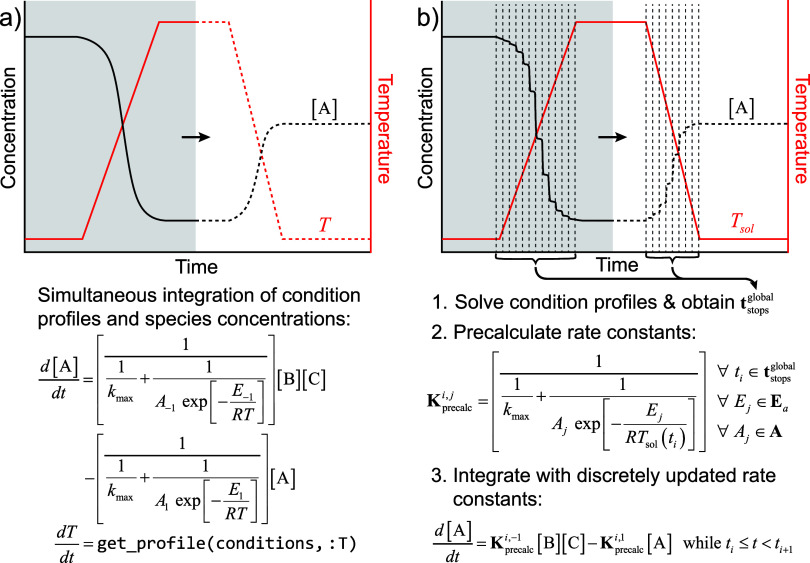
Differences between the two variable-rate kinetic simulation methods in *Kinetica*. (a) The continuous rate update formalism embeds the functional form of the rate constant of every reaction into the compiled RRE, allowing reaction rates to evolve continuously with experimental conditions and time. (b) The discrete rate update formalism precalculates rate constants at discrete time points using interpolated solutions of condition profiles—here *T*_sol_ represents a solution of a temperature profile. During a kinetic simulation, the rate constants are updated at these time points to simplify RRE compilation.

Combining these equations with the generated RRE forms another system of DAEs. DAEs are less easily solved by numerical methods than ODEs, so we use *ModelingToolkit* to automatically perform a set of substitutions that turn the DAEs back into ODEs. In this case, the substitution is simple—each rate constant equation (defined within the selected kinetic calculator) gets directly substituted into the relevant locations inside the RRE. Using a ConditionSet with only variable temperature and the PrecalculatedArrheniusCalculator, the first ODE in our toy CRN above therefore becomes
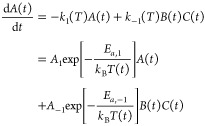
9Note that the resulting ODE is purely a function of time. We perform this substitution for the entire RRE to obtain a fully condition-dependent system of ODEs, which describes the evolution of the CRN with time and all the continuously variable conditions in the ConditionSet (Figure 6a).

#### 3.2.2 Performance Considerations

Solving stiff systems of ODEs, such as those created by complex CRNs, is an inherently multiscale problem. Some reactions, including many bond-breaking events, have large activation energies, leading to very small rate constants and very slow reactions. Other reactions, such as radical recombinations, can be barrierless processes, which occur as frequently as the collisions between reactants.

*DifferentialEquations.jl* contains implementations of many solvers across a number of programming languages, so the choice of solver is key to achieving efficient kinetic simulations. We recommend using the CVODE_BDF solver from the *Sundials* suite.^43^ It is an implicit adaptive-order multistep VODE (variable-coefficient ODE) solver that can handle both large ODE systems and very high stiffness, and it has performed best for the systems considered here.

In recent years, there has been significant research into performing high-efficiency calculations on stiff systems such as CRN RREs by using methods other than implicit VODE solvers.^44,45^ Indeed, simulations using implicit VODE solvers can suffer from high computational expense due to the complexity of numerical Jacobian evaluation and decomposition in each time step.

Most of these methods focus on ways of partitioning the full CRN into groups of reactions with different time scales, effectively removing the stiffness from the ODE system. This is the foundation of methods such as the quasi-steady state approximation,^46^ computational singular perturbation^47^ and the intrinsic low dimensional manifold,^48^ which utilize the Jacobian of the system to identify groups of reactions that can be separated by time scale. This, however, would be unfeasible for variable conditions—species concentrations and rate constants are both time-dependent, so the Jacobian of the system must be diagonalized often to determine which reactions should be separated. This can be viable for small CRNs where these operations are relatively inexpensive, but repeated diagonalization of large Jacobians would incur significant computational expense.

Alternate methods exist for instead estimating the characteristic time scales of reactions by less expensive means, making explicit multitime-scale solutions of large CRNs computationally cheaper to obtain than their respective implicit VODE solutions.^49^ However, since we symbolically model the CRN within *ModelingToolkit.jl* and can therefore construct analytic Jacobians that take advantage of sparse linear algebra routines, we can greatly increase ODE solution speed without needing to perform any explicit CRN separation.^50^

Despite the efficiency of the ODE solution algorithms in *Kinetica*, the extremes of both fast and slow reaction types can still cause problems during numerical integration. We therefore implement several additional performance optimizations, as follows.

##### 3.2.2.1 Handling Slow Reactions

Some reactions proceed so slowly that, even at high reactant concentrations, the resulting product concentrations will be negligible over the time scale of the simulation. These reactions can simply be removed from the CRN to maximize efficiency of the ODESystem compilation.

We assume a reaction *i* can be removed from the CRN if, over the entire time scale of the simulation, the maximum rate of that reaction *r*_*i*_^max^ is smaller than the precision, *rtol*, of the ODE solver
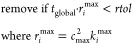
10where *t*_global_ is the global simulation time (see below), *k*_*i*_^max^ is the rate constant for the reaction at the maximum temperature that will be seen within a simulation, and *c*_max_ is a maximum concentration. This concentration should be a value that is greater than the maximum concentration any species in the reaction mixture should obtain. We recommend setting this at 2–5 times the concentration of the initial reactants. This ensures that any reactions that are removed from the CRN would never be formed in quantities greater than the numerical noise in the simulation, below which results should not be considered to be accurate.

##### 3.4 Handling Fast Reactions

Very fast reactions are more problematic to kinetic propagation. The faster a reaction proceeds, the smaller the required time step of the ODE solver. If the time step is very small, many timesteps have to be taken to numerically integrate the system. In some cases, fast reactions may demand that the time step falls to such a small value that it is less than the smallest precision ϵ that is computationally representable by the global simulation time *t*_global_. This causes floating point underflow to occur when this time step is added to the simulation time, resulting in the simulation time not advancing.

We typically represent *t*_global_ with an IEEE 64-bit floating point number (referred to hereafter as a Float64) where ϵ = 2^–53^ ≃ 10^–16^. The smallest value that can be added to such a number is therefore *t*_global_ϵ. Simulation time accumulation therefore fails when.

(a) *t*_global_ is so large that the time step being added to it is less than *t*_global_ϵ,
(b) the simulation conditions are so favorable to reactions that the time step must fall below *t*_global_ϵ to resolve fast reactions,
(c) a combination of the above occurs.

This problem is therefore intrinsic to long-time-scale kinetic simulations. While calculations with numeric types of greater precision are possible, very few sparse matrix solvers are designed to perform operations on numeric types other than Float64, and those that can handle arbitrary numeric types are currently too inefficient to enable adequate performance.

*Kinetica* tackles the problem of fast reactions on two fronts. First, we allow for limiting the maximum rate constant. In a fluid, the maximum possible frequency of collision is limited by the diffusion rate. We can therefore use partial diffusion control to place a hard limit on the maximum rate.^51^ In diffusion-controlled reactions, the rate constant *k*_D_ is
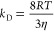
11where η is the viscosity of the fluid at temperature *T*. Partial diffusion control limits the total rate of reaction *k*_*r*_ as follows
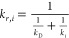
12This ensures a smooth transition between the calculated rate constants and diffusion control.

However, even with a maximum rate constant in place, some combinations of variable conditions and overall time scale can still lead to floating point underflow during propagation. We sidestep this issue with a multitime scale approach to simulation time accumulation. In this approach, a single simulation can be split up into chunks of simulation time, each of length τ_c_. When a kinetic simulation is initialized with species concentrations **c** = **c**_0_, global simulation time *t*_global_ = 0.0, local (chunk) simulation time *t*_local_ = 0.0, and number of chunks *n*_c_ = 0, it is only allowed to proceed until *t*_local_ = τ_c_, at which point the simulation pauses. The solver is then reinitialized at *t*_local_ = 0.0 with **c** = **c**_final_, where **c**_final_ is the vector of concentrations at the end of the previous chunk, and *n*_c_ is incremented.

This approach is continued with
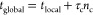
13until *t*_global_ reaches the end of the global simulation time *t*_end_. By replacing *t*_global_ with the smaller *t*_local_ during time step accumulation, we allow small timesteps to be taken down to the value of *t*_local_ϵ. This lets us arbitrarily extend the numeric precision of simulations, ensuring we avoid floating point underflow due to fast reactions and long time scales.

#### 3.2.3 Disadvantages of Continuous Variable Conditions

The above performance enhancements, along with a careful choice of ODE solver from *DifferentialEquations.jl*, can enable highly efficient ODE solutions over long time scales under challenging environmental conditions (such as high temperatures, which exacerbate the stiffness of generated ODE systems). However, the continuously variable kinetic formalism has two major disadvantages.

First, to compile a continuous analytical RRE that is dependent on environmental conditions, rate constants for every reaction must be expressible purely as a function of those conditions. This limits the level of theory that can be used to describe rate constants, as theories beyond the standard (harmonic/rigid-rotor) transition state theory (TST) approach are too complex to be expressed as continuous functions of external conditions. The continuously variable kinetics approach is therefore currently limited to an Arrhenius theory-based rate expression.

Second, even when using analytically expressible rate constants with simple functional forms, compiling high-performance functions from symbolic expressions at runtime comes with a high computational cost. Each ODE in the resulting ODESystem can contain many terms, each with their own rate constants, which must be expanded out to obtain their full functional forms. This leads to an ODESystem that can take several hours to compile, which is problematic in the context of the iterative CRN exploration algorithm detailed in Section 2.2.2, where many CRNs must be compiled and solved to reach convergence. This is made even worse by the poor scaling of compilation time with CRN size, whereby every new species adds an ODE to the ODESystem and reactions involving such species can appear in many other species’ ODEs. All new reactions come with rate constants that must similarly be expanded, causing compilation time to increase dramatically.

### 3.3 Simulations with Discrete Variable Conditions

For the reasons noted above, it is often impractical to use the continuous variable kinetic formalism when solving for the long-time-scale kinetics of molecular degradation within large CRNs. We therefore introduce an approximation to this formalism that dramatically cuts down on compilation time with negligible loss in solution accuracy (summarized in Figure 6b).

Compilation time for RREs with static kinetics is a fraction of that of their continuous variable kinetic counterparts. This is due both to the expansion of every rate constant expression required to form the variable kinetic ODESystem and also to the symbolic analytic partial differentiation that must occur on these complex ODESystems to obtain their analytic Jacobians. As static kinetic RREs only have a single parameter for each rate constant, there is no such expansion and their analytic Jacobians are far simpler to calculate.

These parametric rate constants are usually set at the beginning of a static kinetic simulation and left as such. However, it is possible to modify their values at runtime by using callbacks within the solution of the ODE. *DifferentialEquations.jl* features a rich callback library that facilitates such modifications, allowing us to discretely update rate constants on a finite grid of time points throughout a simulation.

The method begins with the ConditionSet, where users can set a rate constant update time step τ_r_. This is used to generate an array of stopping points **t**_**stops**_ for each variable condition profile along the global simulation timespan, although checks are in place to ensure that no unnecessary stops are made during times when a condition is constant
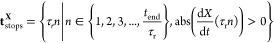
14where *t*_end_ is the global simulation end-time and *X* is an arbitrary variable condition. Once **t**_stops_^**X**^ has been calculated for each variable condition profile, the arrays are combined into a single set of stopping points for the whole ConditionSet, **t**_stops_^global^.

When the discrete kinetic solver is called, the variable condition profiles in the ConditionSet are each independently solved over the simulation timespan, decoupled from the main RRE. This reduces the stiffness of the main RRE, and allows us to precalculate the value of each variable condition at the times within **t**_stops_^global^ using *DifferentialEquations.jl*’s solution interpolation. These values are used within the kinetic calculator to calculate the reaction rates at each stopping point, forming a matrix of precalculated rates **K**_**precalc**_. We provide example code showing how **K**_**precalc**_ is populated in Supporting Information Figure S2.

During RRE solution, every time point in **t**_stops_^global^ is used as a time-stepping milestone. Once the solver reaches a time point *i* within **t**_stops_^global^, a callback modifies its internal state by replacing its parameters **k** with the precalculated rate constants for that time stop, **K**_precalc_^**i**^. The simulation then continues with these rate constants until the next time within **t**_stops_^global^ is reached, at which point the callback is repeated.

By discretely updating rate constants on a fine enough τ_r_ grid, it is possible to recover an approximate concentration–time profile for every species in a given CRN with negligible error when compared to the result generated by the continuous formalism in Section 3.2.1. We show that this approximation holds while the gradients of the condition profiles are locally constant in Section S2 of the Supporting Information. Furthermore, since the compiled ODESystem takes a form closer to the static kinetic RRE in eq 5 rather than the continuous formalism RRE in eq 9, CRN compilation time is dramatically reduced. Full results are discussed in Section 4.

While compilation time is greatly improved under the discrete formalism, we still must also maintain the previously discussed solver optimizations to keep solution times relatively low. This is simple for all optimizations discussed, apart from the multitime scale approach to simulation time accumulation in Section 3.2.2. For this to coexist with the discrete formalism, care must be taken to convert the global-time **t**_stops_^global^ to local chunk-time values. This is done using a variation of eq 13
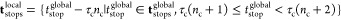
15

This selects the global time stops that lie within the current simulation chunk’s timespan and converts them into time stops which can be used on the local timespan.

The decoupling of variable rate constant calculation from the main RRE is a vital advantage of the discrete formalism employed here, as it allows for almost any level of rate constant calculation to take place without interrupting the overall CRN solution (provided the rate constants are not themselves dependent on midsolution quantities such as species concentration). For example, it would be possible to precalculate DFT-level TST rate constants based on the experimental conditions requested and to use them on-the-fly within discrete kinetic simulations that mirror the prohibitively expensive exact continuous kinetic simulations at this level.

#### 3.3.1 Disadvantages of Discrete Variable Conditions

The discrete kinetic formalism has many benefits, but should not always be used under every circumstance. Changing the rate constants at discrete intervals introduces discontinuities in the concentration gradient of every species simultaneously. These can typically be readily handled by ODE solvers that are capable of adaptive time-stepping, but such solvers must advance simulation time very carefully to maintain numerical stability. This can sometimes slow the overall solution of CRNs compared to the continuous formalism, depending on the severity of the discontinuities. The solution cost of discrete formalism simulations increases as τ_r_ is decreased, as this necessitates more stopping points where the callback is run, and therefore more gradient discontinuities. Conversely, τ_r_ can be increased to reduce the number of stopping points, although this can make the concentration gradient discontinuities at these points larger and decrease the numerical stability of the solution.

The overall computational time-savings from discrete formalism compilation usually vastly outweigh the potential small performance degradation to solution time. However, one may envisage two situations in which this advantage is not realized.

(a) An exact, smooth solution to a given RRE is required,
(b) the gradient discontinuities introduced by the discrete formalism are so severe that solution time is being impacted and the RRE is being repeatedly rerun, for example, as part of an ensemble calculation. In this case, the additional cost of continuous formalism compilation may be outweighed by the savings from continuous formalism solving as the ODESystem only needs to be compiled once and can be resolved many times.

Outside of these situations though, the discrete variable kinetics formalism can be used to greatly accelerate kinetic modeling of generated CRNs; this, as well as the advantages of iterative CRN construction, is demonstrated in the case study below.

## 4 Case Study

To showcase the functionality implemented in the *Kinetica* packages, we study the CRN and kinetics of ethane pyrolysis under a variable temperature profile. There are a wealth of experimental and theoretical results pertaining to this problem under a variety of variable experimental conditions that make this an excellent system for benchmarking the performance and capabilities of our methods.^13,52,53^ In particular, we aim to replicate some of the experimental pyrolysis results of Xu et al.,^54^ who performed a series of pyrolysis experiments in a tubular quartz flow reactor at temperatures ranging from 550 to 850 °C, before analyzing the pyrolysis products with a combination of gas chromatography, thermal conductivity detection, flame ionization detection, and mass spectroscopy. These results are particularly interesting because they include measured temperature profiles along the center line of the flow reactor, which we can replicate with our methodology.

In all of the following results, we employ the *KineticaKPM.jl* modular kinetic calculator, which uses ML to predict reaction activation energies on-the-fly with TST. In the demonstrative calculations reported here, we employ simple collision theory to approximate the TST rate of reaction. Here, the temperature-dependent rate of a bimolecular reaction between chemical species *A* and *B* is defined as
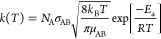
16where *N*_A_ is Avogadro’s number,  is the collision cross section of the reactants (where *r*_A_ and *r*_B_ are the hard sphere radii of the collision partners), *k*_B_ is Boltzmann’s constant, *T* is the variable simulation temperature, μ_AB_ is the reduced mass, *E*_a_ is the activation energy, and *R* is the ideal gas constant.

The key parameter is this approximation is the activation energy *E*_a_. In order to estimate *E*_a_ for sampled reactions, we employ a previously reported ML framework that predicts *E*_a_ given structures for the reactants and products of a given elementary chemical reaction.^33^ A detailed comparison of the results of our kinetic simulations with experimental pyrolysis results will be presented in an upcoming paper; here, we focus on comparing and contrasting the aspects of CRN construction (direct and iterative methods) and kinetic simulations (continuous and discrete methods) described above.

### 4.1 Variable Temperature Conditions

To replicate the experimental pyrolysis results in ref (54), a ConditionSet was constructed with simulation temperature bound to a DoubleRampGradientProfile. This is a gradient-based condition profile that defines two linear temperature ramps separated by a plateau. The relevant gradient function is
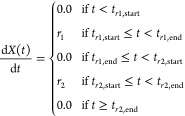
17where *r*_1_ and *r*_2_ are the rates of change of the two linear ramps and *t*_*r*_1_,start_, *t*_*r*_1_,end_, *t*_*r*_2_,start_, and *t*_*r*_2_,end_ are the respective start- and end-times of the first and second ramps, determined by the lengths of the starting, middle, and ending plateaus.

**Figure 7:**
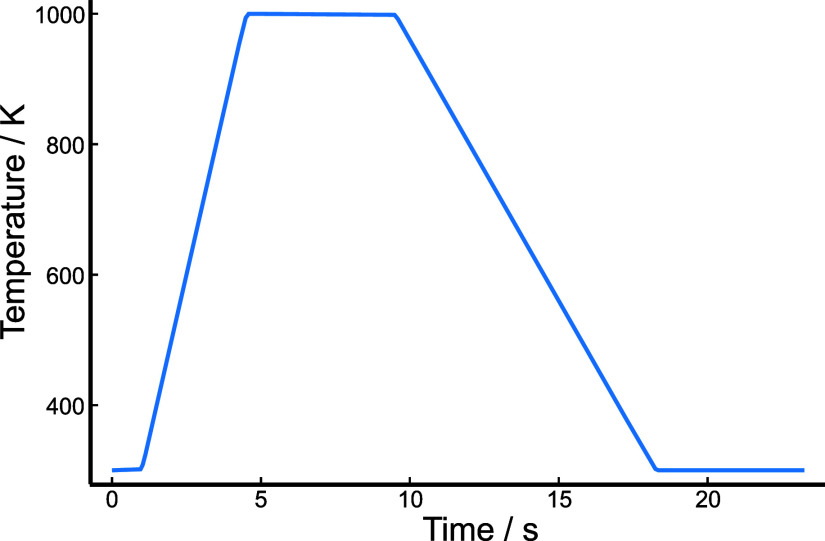
Computational temperature profile matching the 1000 K experimental temperature profile in ref (54), after implementation and solution within *Kinetica.jl*.

By extrapolating the temperature profiles shown along the tubular reactor used in the original work such that the temperature profile starts and ends at 300 K, and by using the reported reactant flow rate to convert distance along the reactor into time, we arrived at the temperature profile shown in Figure 7 to replicate the experimental 725 °C (≈1000 K) pyrolysis profile. The code for the resulting ConditionSet is given in Supporting Information Figure S3.

### 4.2 SE-GDS Simulations

The SE-GDS algorithm requires a library of possible graph moves to apply to an input molecular system to stochastically sample chemical reaction space. Here, we used an exhaustive selection of 2-atom and 3-atom moves to explore as much of the available reaction space as possible. The full library of allowed reactions is available in Supporting Information Figure S4.

Additionally, SE-GDS requires many input parameters. Except for variables controlling how many reactions are generated per SE-GDS run, these parameters are kept constant for the duration of a CRN generation. They are therefore provided alongside the graph move library as an input file, shown in Supporting Information Figure S5.

### 4.3 Results of Direct Exploration and Continuous Kinetics

To perform direct CRN exploration, a system of ethane molecules must be provided to initialize SE-GDS. As high-temperature ethane pyrolysis can create free radical species, the radical chain growth mechanism shown in Figure 3 can occur. The number of molecules in the initial system therefore dictates the maximum size of any species resulting from this mechanism and thus the complexity of the resulting network. This makes convergence with the direct exploration method increasingly difficult as more molecules are added. At the pyrolysis temperatures being modeled here, species with more than four carbon atoms were not experimentally observed. We therefore chose an initial system of 2 ethane molecules to perform CRN exploration using the direct method.

**Figure 8:**
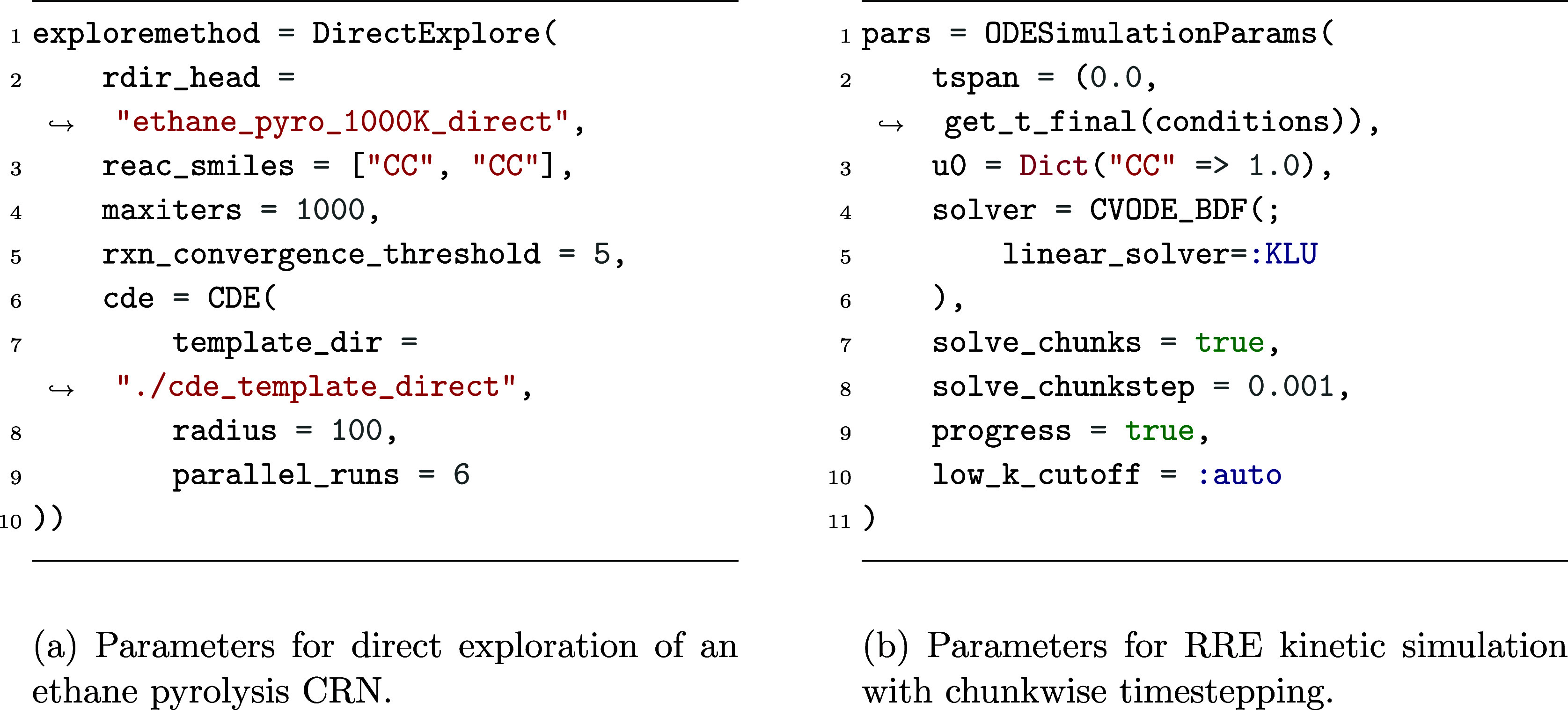
*Kinetica* parameter blocks used within network *C*_4_^D^ exploration and simulation.

In *Kinetica*, this calculation is requested by building a DirectExplore exploration parameter block, shown in Figure 8a. This specifies that two ethane molecules (represented as CC within SMILES notation) should be repeatedly subjected to single-ended mechanism searches of *n*_r_ = 100 (defined by the CDE.radius parameter) until either 10^3^ exploration iterations have elapsed (maxiters) or no new reactions have been found for 5 iterations (rxn_convergence_threshold). This is a reasonable convergence criterion, requiring 3 × 10^3^ reactions to have been sampled without any change to the CRN to signal convergence (noting that each iteration performs 6 concurrent CDE explorations, as set by parallel_runs).

**Figure 9:**
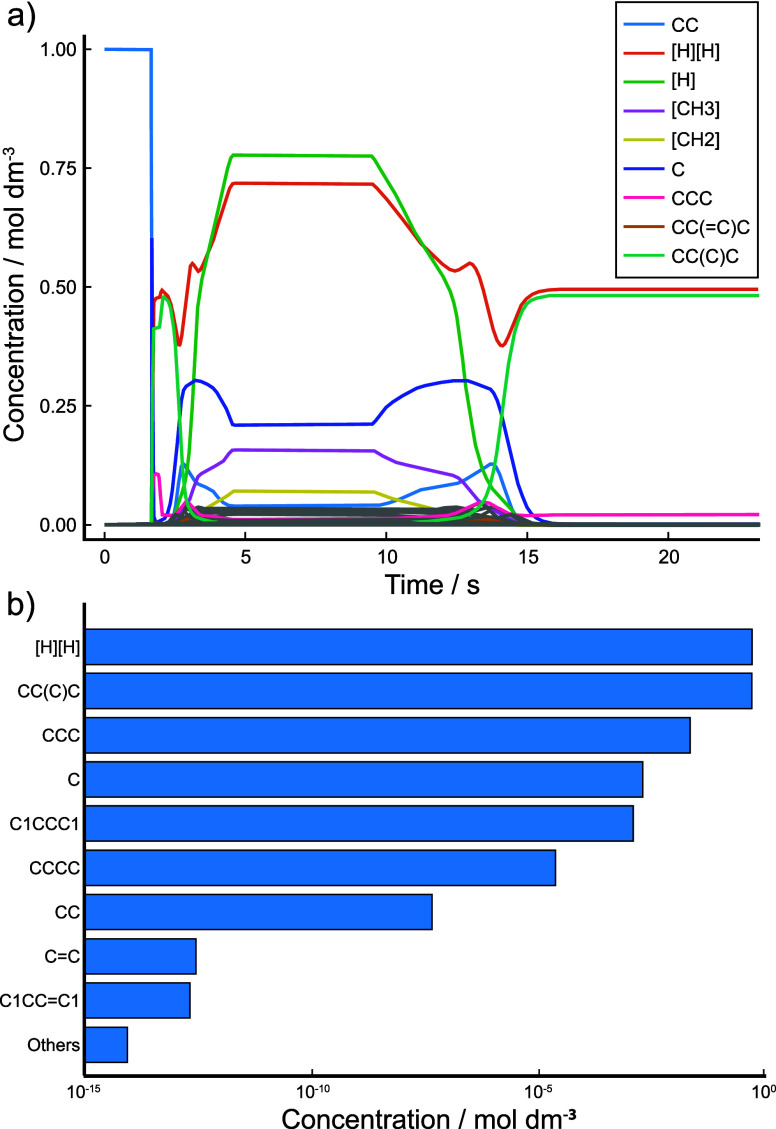
Kinetic simulation results of network *C*_4_^D^ performed with the continuous rate update approach. (a) Concentration–time profiles of prevalent species. (b) Final concentrations of species at simulation end.

This direct exploration resulted in a CRN, which we refer to as network *C*_4_^D^, consisting of 3758 reactions and 164 unique species. We performed a kinetic simulation on this CRN using the continuous rate update formalism with chunkwise time-stepping at τ_c_ = 1 ms. This is requested from *Kinetica* by building an ODESimulationParams parameter block, shown in Figure 8b. The resulting concentration/time profiles are shown in Figure 9a.

As the simulation temperature increases past 450 K, all ethane (CC in SMILES) in the system quickly breaks down, forming methane (C) and larger molecules such as propane (CCC) and isobutane (CC(C)C). The absence of any radical species present in the reaction mixture at this stage indicates preferential recombination into larger species. This conversion to larger species results in fewer hydrogenated carbon atoms on average, noting that diatomic hydrogen ([H][H]) is also released. As temperature increases further, these larger species also become unstable and break down into methane, methyl radicals ([CH3]) and diradicals ([CH2]), and ethane. Hydrogen atoms ([H]) are also released in large quantities at higher temperatures. Once the system temperature reaches 1000 K and reaches a plateau, a steady state is quickly formed within the CRN, analogous to a kinetic simulation with static conditions.

Once the temperature starts to decrease again, both single-atom and molecular hydrogen recombine with other species in the system to form stable hydrocarbons such as methane, ethane, and propane. Past 725 K, however, this behavior changes toward creating isobutane as the kinetic rate equations being used identify it as the most kinetically accessible species at ambient temperatures. This leaves nearly all carbon atoms in isobutane molecules at the end of the simulation as well as the molecular hydrogen that is released as a result. Final species concentrations are shown in Figure 9b.

To demonstrate the poor scaling of the direct exploration method with initial system size, we repeated this exploration with an additional ethane molecule in the starting system, allowing for species containing up to six carbon atoms to be formed. While we do not expect these new species to be formed under the simulation conditions, the direct exploration method does not make any attempt to selectively explore chemical reaction space, instead attempting to find all possible reactions within a given reactive radius *n*_r_.

This change, while seemingly small, had a profound impact on the resulting CRN (labeled network *C*_6_^D^). Exploration into the newly accessible areas of chemical space allowed more than 5000 species to become accessible for reactions, over an order of magnitude larger than the number of species discovered in the CRN comprising just two ethane molecules. The combinatorial nature of CRNs revealed an even larger reaction space, with over 1.34 × 10^5^ reactions being discovered before exploration was stopped by an error. This error was found to be the result of a species being impossible to rationalize into SMILES, another problem associated with uncontrolled exploration into kinetically inaccessible and uncharted chemical space.

Even if convergence of network *C*_6_^D^ were possible, CRNs of this size are too expensive to compile into solvable ODESystems under the continuous kinetic formalism described in Section 3.2.1; in addition, such CRNs are also extremely inefficient to solve as many reactive pathways are kinetically irrelevant.

### 4.4 Validating the Discrete Kinetic Approximation

While the solution for the kinetic simulation of network *C*_4_^D^ above was obtainable, the compilation time (and associated memory cost) of such a large network was very high. This and the much larger network *C*_6_^D^ are therefore ideal benchmarks for the discrete rate-update formalism, as it dramatically reduces this compilation time and makes otherwise inaccessible results obtainable.

**Figure 10:**
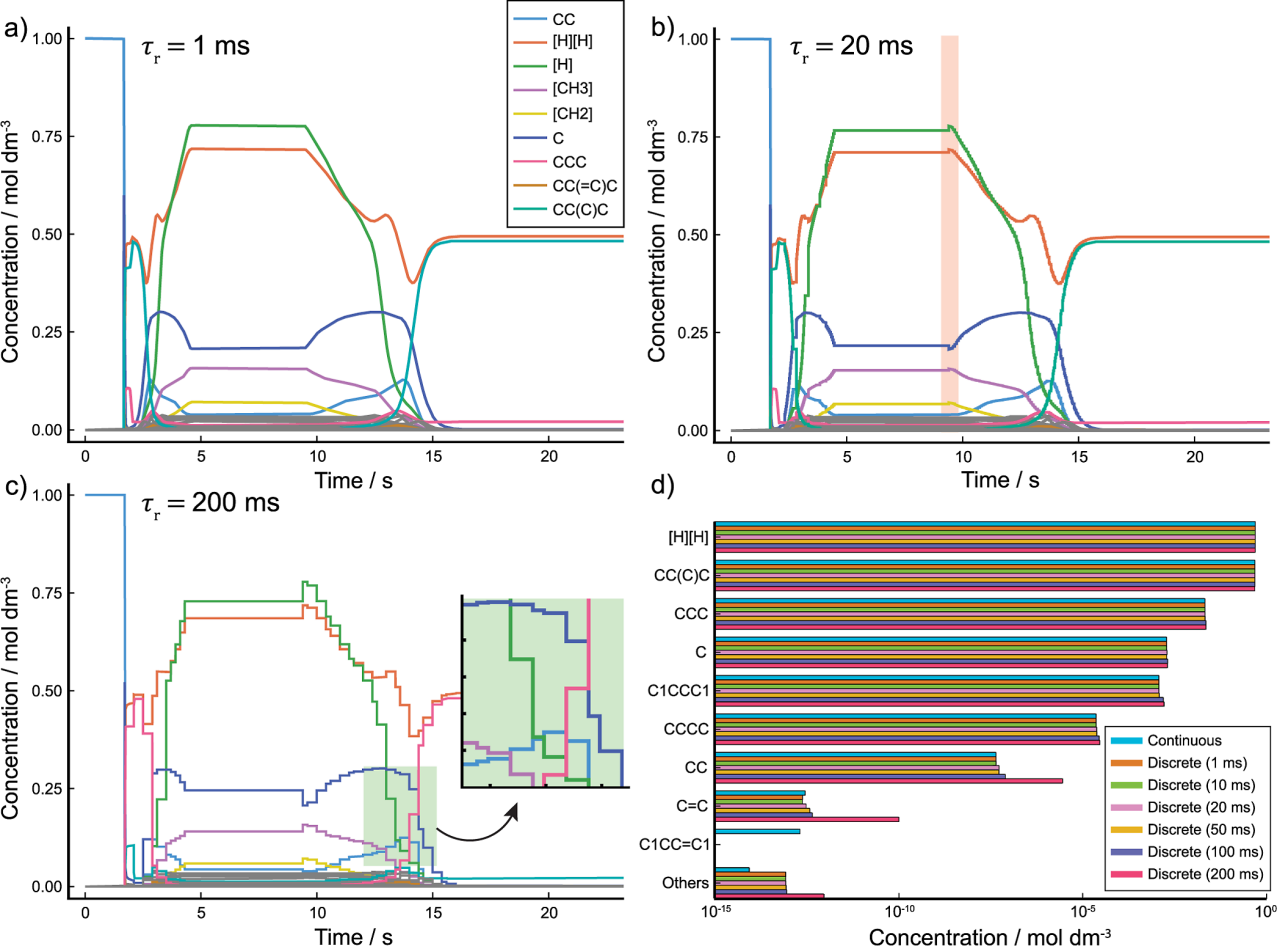
Kinetic simulation results of network *C*_4_^D^ performed with the discrete rate update formalism. (a) Concentration–time profiles with τ_r_ = 1 ms. (b) Concentration–time profiles with τ_r_ = 20 ms, with concentration anomalies when simulation temperature decreases highlighted. (c) Concentration–time profiles with τ_r_ = 200 ms. (d) Comparison between final concentrations of species at the end of continuous and discrete rate update simulations with varying values of τ_r_.

We first establish that the discrete formalism is a good approximation of fully continuous variable kinetics by rerunning the kinetic simulation of network *C*_4_^D^ with discretely updated rate constants. Initially, we update rate constants every τ_r_ = 20 ms. The results of this simulation are shown in Figure 10b.

The τ_r_ = 20 ms discrete approximation results show a high degree of accuracy with respect to the previous continuous formalism results, in terms of both the final species concentrations at the end of the simulation and the concentration profiles of individual species observed throughout the simulation. The only exception to this is just before *t* = 10 s, where the simulation temperature begins to decrease and species concentrations briefly jump to values not observed in the continuous formalism results (marked by a red box in Figure 10b). This is due to the temperature gradient not being locally constant for a short period of time while a discrete rate constant update is taking place.

A direct comparison between the final species concentrations is shown in Figure 10d, where we show that even on a logarithmic scale, the discrete formalism can produce an extremely close match with the continuous formalism results. The exception to this is for species with very small final concentrations, although these species fall into the domain of numerical noise within the simulation and are not guaranteed to be accurate under either rate update formalism. These results were obtained in a significantly shorter time than the continuous formalism results. The RRE compilation time for the kinetic simulation was reduced by more than an order of magnitude, with the RRE solution time being reduced by 55%. Full results are shown in Table 1.

**Table 1:** Timings for Kinetic Simulation of Network *C*_4_^D^ under Continuous Rate Update Formalism and under Discrete Rate Update Formalism with Varying τ_r_

rate update formalism	τ_r_	compile time (s)	simulation time (s)
continuous	N/A	5169	326
discrete	1 ms	445	240
discrete	5 ms	449	163
discrete	10 ms	450	151
discrete	20 ms	451	145
discrete	50 ms	451	140
discrete	100 ms	452	134
discrete	200 ms	452	135

Although τ_r_ = 20 ms was initially chosen as the discrete rate update time step, we can potentially reduce the solution time of the discrete formalism RRE further by increasing this value. This increases the time between discrete rate constant updates, potentially decreasing the cost of performing these updates. However, this has to be balanced carefully, as increasing τ_r_ also increases the relative size of the gradient discontinuities being introduced into the RRE at each update point. These discontinuities require careful adaptive time-stepping to resolve, potentially increasing the solution time of the RRE. The effects of modifying τ_r_ on the simulation time are also shown in Table 1, with select kinetic profiles at other values of τ_r_ in Figure 10a,c. Final species concentrations of these simulations are also shown in Figure 10d.

For the CRN studied here, we only observed a decrease in RRE solution time as τ_r_ was increased (within the margin of error). However, with other CRNs and variable condition profiles we have seen solution times increase dramatically at higher values of τ_r_ due to increased numerical instability surrounding the gradient discontinuities. Regardless, while increasing τ_r_ significantly can improve solution time, we do not recommend doing so as it becomes very difficult to follow the resulting species concentration profiles and makes simulations prone to numerical error with diminishing gains in solution time. This is especially evident in the low final concentration regime (Figure 10d), where the results with τ_r_ = 200 ms diverge considerably from those with smaller values of τ_r_.

At large values of τ_r_, concentration profiles behave in an almost stepwise manner. By zooming in on these concentration steps as in Figure 10c, we can see that the steps are actually very fast changes in concentration, followed by equilibration to a steady state, which lasts until the next rate constant update. This behavior is intuitive when we recall that, between each rate constant update, the RRE has static kinetics and will therefore reach a steady state due to the principle of detailed balance.

**Figure 11:**
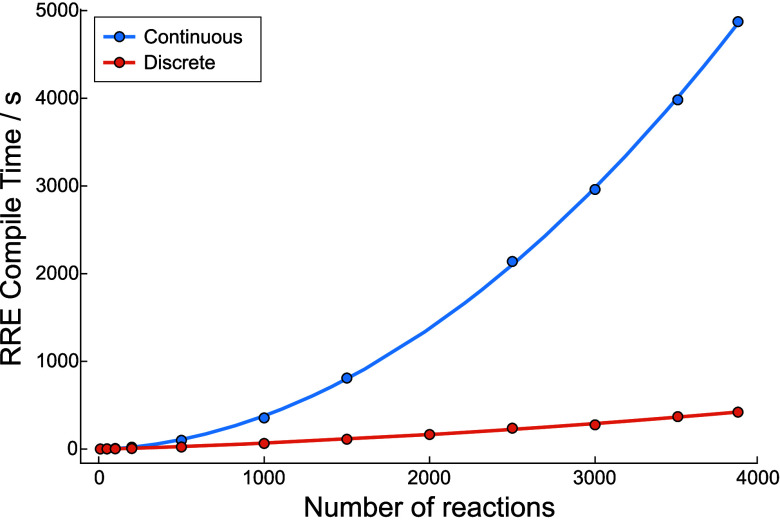
Scaling curves for RRE compilation time under continuous and discrete rate update methods, fit with second-order polynomials.

We have also investigated the computational scaling of RRE compile time under both rate update formalisms with CRN size by compiling successively larger subnetworks of *C*_4_^D^. These results are shown in Figure 11, where a polynomial function was fitted to each set of compile times to enable predictive extrapolation. The continuous kinetic formalism is shown to scale very poorly with CRN size, while the direct kinetic formalism demonstrates much more favorable scaling behavior.

Using these polynomial fits, we can approximate how long it would take to compile network *C*_6_^D^ in its current state. Under the discrete formalism it would take approximately 72 h, which, while a significant amount of time, would not be unobtainable if necessary. However, under the continuous formalism, we estimate this RRE would take over 60 days to compile with the current methodology. While this may look fairly dire, we again emphasize that this is one of the expected downfalls of CRN exploration with the direct method, while a more directed approach to exploration of chemical reaction space would remove the need for RREs of this size by reducing the number of reactions considered.

Since the discrete rate update formalism introduces negligible error and greatly expedites the compile time and solve time of RREs (provided a reasonable value of τ_r_ is set), all further kinetic simulations in this work will be performed using this strategy. In particular, we use τ_r_ = 10 ms as this removes the previously discussed artifacts observed when τ_r_ = 20 ms and provides excellent accuracy while retaining a fast RRE solution time.

### 4.5 Iterative Exploration

To resolve the systematic under-sampling of generated species in the direct exploration CRN, we repeated the CRN exploration using the iterative exploration method. This method propagates exploration along kinetically accessible reaction pathways, ensuring that species which exist at high concentrations are reacted and fully sampled under the given simulation conditions.

**Figure 12:**
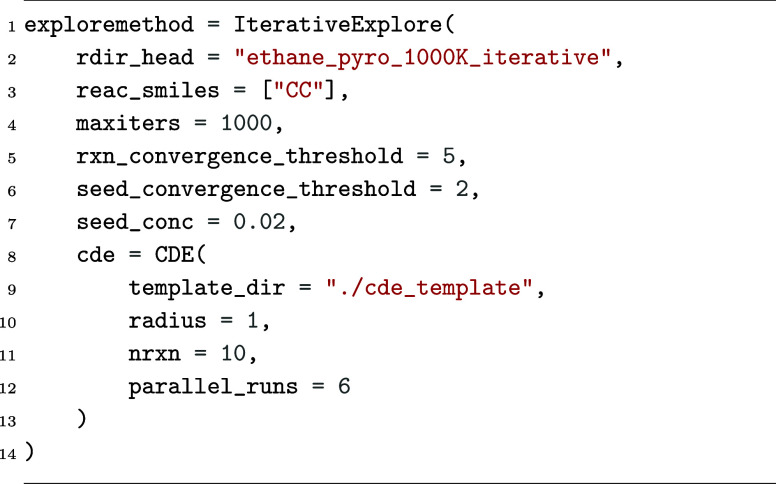
*Kinetica* parameters for iterative exploration used within network *C*_4_^I,0.02^.

When performing an iterative exploration, the initial system needs to only contain one of each of the possible initial reactants, as other systems of high concentration seed species are created by *Kinetica* as exploration continues. There is therefore only one ethane molecule requested for reaction in the IterativeExplore exploration parameters shown in Figure 12. This parameter block has many of the same options as the simpler DirectExplore, except for a few additions.

The most important of these new parameters is the seed selection concentration cutoff *c*_select_ (seed_conc), which controls the concentration at which species are selected to be in the seed system at each iterative level of the CRN. Setting this parameter too high results in too few species being selected to undergo further reactions each level, leading to a different kind of under-sampling—*kinetic* under-sampling. This occurs when the CRN is systematically fully sampled (that is, *Kinetica* has explored the CRN until it cannot find any more reactions given the seed species available for reaction), but there are still kinetically important species that remain under-sampled because they have not been selected as seeds. However, setting *c*_select_ too low can result in too many seeds being selected for further reactions at each level, making the resulting CRN extremely large. Also of note is the seed_convergence_threshold parameter, which dictates how many levels of exploration must be completed with no change in the next level’s seed system in order for the network to be considered converged. Usually this can be set to two; after two iterations of exploring reactions exclusively between the same species, the chance of finding any further kinetically important reactions is very low.

We began by running an iterative CRN exploration with *c*_select_ = 0.02 mol dm^–3^. This network was also limited to exploring species containing up to four carbon atoms, but this cannot be achieved by limiting the size of the initial molecular system as in the direct exploration method because the iterative method creates new systems of larger molecules as it proceeds. We therefore use *Kinetica.jl*’s RxFilter option to remove reactions resulting in species with greater than four carbon atoms each time a kinetic simulation is run. Further details on how this is implemented are given in Section S3.4 of the Supporting Information.

We ran the iterative CRN exploration to create network *C*_4_^I,0.02^. This network required nine levels of iterative exploration to converge, finishing with a network of 3425 reactions and 104 unique species. This is notably smaller than network *C*_4_^D^ in both reactions and species, and when running a kinetic simulation on the final network, the results are markedly different. This is due to network *C*_4_^I,0.02^ being kinetically under-converged because kinetically important reactions have not been found due to *c*_select_ being set too high.

We therefore performed another iterative CRN exploration with *c*_select_ = 0.01 mol dm^–3^ to obtain better kinetic convergence. This resulted in network *C*_4_^I,0.01^, which took 11 levels of exploration to converge, with 8026 reactions and 130 species. This is over double the number of reactions explored in *C*_4_^D^, but fewer species overall. This occurs because the iterative exploration method never explores species with kinetically inaccessible precursors; as a result, this network is therefore much more thoroughly explored than *C*_4_^D^.

**Figure 13:**
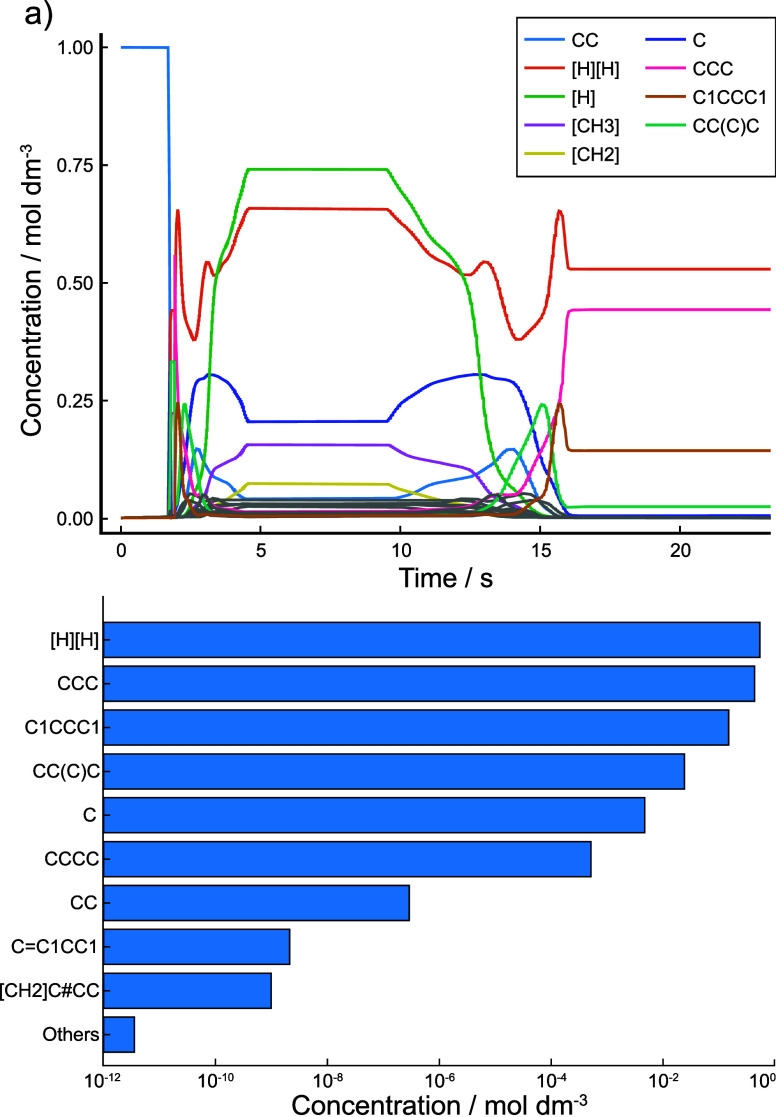
Kinetic simulation results of network *C*_4_^I,0.01^ performed with the discrete rate update formalism at τ_r_ = 20 ms. (a) Concentration–time profiles of prevalent species. (b) Final concentrations of species at the end of simulation.

The kinetic simulation results of network *C*_4_^I,0.01^ are shown in Figure 13. While there are many similarities to the kinetic simulation of network *C*_4_^D^, there are also some differences that clearly demonstrate the presence of other kinetically important reactions. Of note are concentration profiles of propane, isobutane, and cyclobutane (C1CCC1), the latter of which was found by the direct CRN exploration but never emphasized in the kinetic simulation of *C*_4_^D^ due to missing reactions.

These additional reactions are mostly observed during the temperature ramps. During heating, a large amount of cyclobutane is temporarily formed, releasing molecular hydrogen, before breaking down as the temperature continues to increase. During cooling, the rise in concentration of isobutane is halted by increasing concentrations of propane and cyclobutane, leading to the latter being the majority products instead of the former. This is accompanied by an increased final concentration of molecular hydrogen, which is released by the formation of cyclobutane before being consumed by formation of the less substituted propane.

Finally, as with the direct exploration method example, we increased the carbon atom limit in the RxFilter to six and ran another iterative CRN exploration with *c*_select_ = 0.02 mol dm^–3^, forming network *C*_6_^I,0.02^. After 18 levels of exploration, this produced a converged CRN of 14,274 reactions over 707 species. The kinetic simulation results of this network are easily obtainable with the discrete rate update formalism, but they reveal problematic trends.

While not necessarily kinetically converged, the main final product after kinetic simulation of *C*_6_^I,0.02^ is methyl-cyclopentane, a species containing 6 carbon atoms. We know from the experimental reference results that such species should not be present in the final reaction mixture of an ethane pyrolysis at this temperature, indicating that some of the kinetic rate constants being employed are potentially inaccurate. This is due in part to a missing entropic contribution within the rate constants calculated by *KineticaKPM.jl*, which will be the main focus of an upcoming follow-up paper on the possibility of calculating rate constants on-the-fly using machine learned activation energies.

## 5 Conclusions

In this article, we presented *Kinetica.jl*, a Julia package for automated exploration and kinetic simulation of CRNs under arbitrary variable conditions. In particular, we have discussed many of the features of *Kinetica.jl*, including an iterative method for kinetics-guided exploration of chemical reaction space, a modular kinetic calculator interface allowing symbolic condition profiles to be bound to user-defined rate expressions, and a discrete approximation of continuously variable rate constants that greatly accelerates simulations with negligible loss in accuracy.

By combining these elements into a unified workflow engine powered by the SciML ecosystem^30^ and CDE’s new SE-GDS algorithm, defined here, we are able to quickly and efficiently generate large, complex CRNs that are tailored to specific environments, unlocking a theoretical insight into complex degradation processes. Through a multitime scale approach to ODE solution within kinetic simulations, we are able to extend this insight into challenging environmental conditions and long time scales. *Kinetica* has been written with extensibility at its core, allowing users to implement custom ways of calculating rate constants, which can vary with user-defined experimental conditions. *KineticaKPM.jl* is one such extension, providing a ML-based kinetic calculator that allows for fast solution of temperature-dependent RREs. The details of this calculator and intricacies of machine-learned rate constants will be the subject of future work.

We have demonstrated *Kinetica*’s CRN generation, kinetic simulation, and analysis workflow by assembling a number of CRNs for the 1000 K pyrolysis of ethane, with a variable temperature profile set to match an experimental temperature profile measured along the length of a tubular flow reactor. By performing kinetic simulations on the resulting networks, we have demonstrated the utility of the discrete kinetic formalism we employ and highlighted the importance of selective exploration within combinatorially large chemical reaction spaces. Without iterative guidance by kinetic simulation results, it can be near impossible to obtain CRNs that fully characterize all the possible reaction pathways under a set of variable experimental conditions. With this guidance, however, CRNs can become both more accurate and more efficient to solve, which will be vital if automated long-time-scale chemical breakdown simulations are to inform experimental degradation studies.

The iterative exploration method does come with an associated cost. Not only does it have to spend more time thoroughly exploring accessible chemical space but it also requires repeated kinetic simulations to guide the direction of exploration. The former problem could be addressed by modifying the SE-GDS within CDE to deterministically enumerate all unimolecular and bimolecular reactions between one and two species respectively, rather than stochastically sampling chemical reaction space. This would accelerate CRN exploration further as there would be no repeated exploration of previously discovered reactions, which currently takes up the majority of exploration time while CDE attempts to find every possible reaction. Meanwhile, kinetic simulations could potentially be accelerated further by inclusion of an explicit multitime scale integrator that takes advantage of time scale separation within the RRE, but this has not been explored yet within *Kinetica*.

## Data Availability

*Kinetica.jl* is available on GitHub at https://github.com/Kinetica-jl/Kinetica.jl. *KineticaKPM.jl* is similarly available on GitHub at https://github.com/Kinetica-jl/KineticaKPM.jl. Both packages are installable through the Julia language Pkg package manager. Associated data can be found in the Warwick Research Archive Portal (http://wrap.warwick.ac.uk/185872).

## Supporting Information Available

Representative GDS input files and example code-blocks demonstrating aspects of rate calculations and kinetic simulations (PDF)
