# Supplementary material for: Predicting Long-Time-Scale Kinetics under Variable Experimental Conditions with Kinetica.jl
Source: J Chem Theory Comput. 2024 Jun 3;20(12):5196–214. doi: 10.1021/acs.jctc.4c00333 (PMC11209948; doi:10.1021/acs.jctc.4c00333)
Supplement: Supplementary file 1 — ct4c00333_si_001.pdf [file ct4c00333_si_001.pdf]

Supplementary Information

Predicting long timescale kinetics under variable experimental conditions with Kinetica.jl

Joe Gilkes[1,2], Mark T. Storr[3], Reinhard J. Maurer[1,4], Scott Habershon[1]

[1]Department of Chemistry, University of Warwick, Gibbet Hill Road, CV4 7AL Coventry, UK
[2]EPSRC HetSys Centre for Doctoral Training, University of Warwick, Gibbet Hill Rd, CV4 7AL, Coventry, UK
[3]AWE Aldermaston, Reading, Berkshire, RG7 4PR, UK
[4]Department of Physics, University of Warwick, Gibbet Hill Road, CV4 7AL Coventry, UK UK

Ministry of Defence © Crown Owned Copyright 2024/AWE

S1 Implementation Examples

S1.1 Kinetic Calculators

```julia
# A struct holds all the data that the calculator needs to calculate its rate constants.
mutable struct PrecalculatedArrheniusCalculator{kmType, uType, tType} <: AbstractKineticCalculator
    Ea::Vector{uType}
    A::Vector{uType}
    k_max::kmType
    t_unit::String
    t_mult::tType
end

# Functors of the calculator struct can be called to calculate rate constants with the specified conditions.
function (calc::PrecalculatedArrheniusCalculator{uType, uType, tType})(; T::Number) where {uType, tType}
    k_r = calc.A .* exp.(-calc.Ea / (Constants.R * T)) * Constants.N_A * calc.t_mult
    return 1.0 ./ ((1.0 / calc.k_max) .+ (1.0 ./ k_r))
end

# Lets callers know if a set of symbolic conditions can be used with a calculator.
# This calculator can only handle temperature as a condition, so any other symbolic conditions will make this return false.
function has_conditions(::PrecalculatedArrheniusCalculator, symbols::Vector{Symbol})
    return all([sym in [:T] for sym in symbols])
end
```

Figure S1: Shortened definition of the `PrecalculatedArrheniusCalculator` kinetic calculator in *KineticaCore*.

S1.2 Discrete Formalism Rate Precalculation

```julia
# Accumulate global time stops array from all condition profiles.
tstops = get_tstops(conditions)

sc_symbols, sc_values = get_static_conditions(conditions)
vc_symbols, vc_profiles = get_variable_conditions(conditions)

# Initialise length(tstops) rows of rate constants for all reactions in the CRN.
K_precalc = [zeros(rd.nr) for _ in tstops]

# Iterate over time stops.
for (i, tstop) in enumerate(tstops)
    # Create a mapping between all condition symbols and their respective values/profiles.
    bound_conditions = vcat(
        [symbol => value for (symbol, value) in zip(sc_symbols, sc_values)],
        [symbol => profile(tstop) for (symbol, profile) in zip(vc_symbols, vc_profiles)]
    )

    # Pre-calculate rate constants at each time stop.
    # Elipses separate out bound conditions into keyword arguments to the calculator.
    K_precalc[i] = calculator(; bound_conditions...)
end
```

Figure S2: Rate precalculation procedure for discrete variable kinetics simulations. By binding static conditions to their assigned values and variable conditions to the interpolated values at each `tstop` in $\mathbf{t}_{\mathbf{stops}}^{\mathbf{global}}$, rate constants can be precalculated and inserted as rows in $\mathbf{K}_{\mathbf{precalc}}$.

S2 Discrete rate update formalism proof

For the discrete approximation to hold, the gradient of each rate constant with respect to time in the continuous formalism must be approximately equal to the gradient of each rate constant between two neighbouring rate update points, i.e. for rate update points t_1 and t_2:

$$\frac{dk}{dt} \approx \frac{k(t_2) - k(t_1)}{\Delta t}$$

This applies to rate constants given by the equation

$$k(t) = \frac{1}{\frac{1}{k_{max}} + \frac{1}{N_A \sigma_{AB} \sqrt{\frac{8k_B T(t)}{\pi \mu_{AB}}} \exp\left(-\frac{E_a}{RT(t)}\right)}}$$

There is no continuous analytical expression for $\frac{dT}{dt}$, but it is (usually) locally constant. We will therefore make the approximation that within a local region of the kinetic profile, $\frac{dT}{dt} = \alpha$. For the sake of legibility, we also simplify the rate constant expression by removing all constants except for E_a:

$$k(t) = \sqrt{T} e^{-\frac{E_a}{T}}$$

S2.1 Continuous Gradient

$$\frac{dk}{dt} = \frac{dk}{dT} \cdot \frac{dT}{dt}$$

$$\frac{dk}{dT} = \frac{1}{2T^{\frac{1}{2}}} e^{-\frac{E_a}{T}} + \frac{E_a}{T^{\frac{3}{2}}} e^{-\frac{E_a}{T}}$$

$$= e^{-\frac{E_a}{T}} \left(\frac{1}{2T^{\frac{1}{2}}} + \frac{E_a}{T^{\frac{3}{2}}}\right)$$

$$\frac{dT}{dt} = \alpha$$

$$\therefore \frac{dk}{dt} = \alpha e^{-\frac{E_a}{T}} \left(\frac{1}{2T^{\frac{1}{2}}} + \frac{E_a}{T^{\frac{3}{2}}}\right)$$

S2.2 Discrete Gradient

Assuming $T_1 = T(t_1)$ and $T_2 = T(t_2)$:

$$t_2 = t_1 + \Delta t$$

$$\therefore T_2 = T_1 + \alpha \Delta t$$

The rate constants at the rate update points therefore take the form:

$$k(t_1) = \sqrt{T_1} \cdot e^{-\frac{E}{T_1}}$$

$$k(t_2) = \sqrt{T_1 + \alpha \Delta t} \cdot e^{-\frac{E}{(T_1 + \alpha \Delta t)}}$$

The gradient of the line connecting these two points is therefore:

$$\frac{k(t_2) - k(t_1)}{\Delta t} = \frac{\sqrt{T_1 + \alpha \Delta t} \cdot e^{-\frac{E}{(T_1 + \alpha \Delta t)}} - \sqrt{T_1} \cdot e^{-\frac{E}{T_1}}}{\Delta t}$$

As Δt tends towards zero, i.e. as the rate update timestep gets smaller and the approximation gets better:

$$\begin{aligned}
\lim_{\Delta t \to 0} \frac{k(t_2) - k(t_1)}{\Delta t} &= \frac{\alpha e^{-\frac{E_a}{T_1}} (2E_a + T_1)}{2T_1^{\frac{3}{2}}} \\
&= \alpha e^{-\frac{E_a}{T_1}} \left(\frac{2E_a}{2T_1^{\frac{3}{2}}} + \frac{T_1}{2T_1^{\frac{3}{2}}} \right) \\
&= \alpha e^{-\frac{E_a}{T_1}} \left(\frac{1}{2T_1^{\frac{1}{2}}} + \frac{E_a}{T_1^{\frac{3}{2}}} \right)
\end{aligned}$$

This is equivalent to the continuous formalism's expression for $\frac{dk}{dt}$, provided we accept that $T_1 = T(t)$, which is true within the limit imposed. This potentially also only holds while $\frac{dT}{dt}$ is a constant, although this requires further inspection, e.g. when $T_2 = T_1 + \sin(t_2) - \sin(t_1)$.

S3 Additional information for case study

S3.1 `ConditionSet` for pyrolysis temperature profile

```
conditions = ConditionSet(Dict(
    :T => DoubleRampGradientProfile(;
        X_start = 300.0,
        t_start_plateau = 1.0,
        rate1 = 200.0,
        X_mid = 1000.0,
        t_mid_plateau = 5.0,
        rate2 = -80.0,
        X_end = 300.0,
        t_end_plateau = 5.0,
        t_blend = 0.1
    )),
    tconvert(20.0, "ms", "s")
))
```

Figure S3: Kinetica `ConditionSet` used within case study of 1000 K ethane pyrolysis. Defines a variable temperature profile that starts with a 1 s plateau (`t_start_plateau`) at 300 K (`X_start`), before rising to 1000 K (`X_mid`) at a rate of 200 K/s (`rate1`). Temperature is held at this plateau for 5 s (`t_mid_plateau`) before falling back to 300 K (`X_end`) at a rate of 80 K/s (`rate2`), where temperature is held for 5 s (`t_end_plateau`) until the end of the simulation. A blending time of 0.1 s (`t_blend`) is used to smooth out transitions between condition gradient regimes by linear interpolation. A 20 ms discrete rate update timestep is passed to allow the `ConditionSet` to calculate the time points at which rate constant updates will occur.

S3.2 SE-GDS graph move library

2-atom and 3-atom graph moves for CDE's SE-GDS algorithm used within Kinetica ethane pyrolysis CRN generation. Each graph move is a block starting with `move`, followed by the number of atoms involved in that move n_m (`natom 2` represents a 2-atom move). This is followed by two $n_m \times n_m$ subgraphs; the first represents part of the CM of the current molecule system and the second represents a modification to the first.

This is followed by a `labels` keyword, which is accompanied by n_m elemental symbols. This can be used to constrict the graph move to a specific set of atoms, where each symbol corresponds to its respective atom in the above subgraphs. Elemental symbols can also take `*` as a value, indicating

that any atom which fits the bonding pattern in the subgraph can be used. Finally, an optional `prob` keyword allows for setting a probability of graph move selection. This probability is relative to the other probabilities set within the graph move library.

For example, the first graph move in the below library represents a bond breaking between any two connected atoms. This is defined by a 2-atom move with `labels * *`, so the move is unrestrained by atom types, and only depends on a matching subgraph. This subgraph is defined as two atoms which are connected to each other (a 1 at coordinates (a_1, a_2) in the current CM, where a_1 and a_2 are the indeces of the selected atoms, and also at (a_2, a_1) by symmetry). The move specifies that these 1s should be modified to 0s, disconnecting the two selected atoms, with a relative probability of 0.1 out of a total of 1.6, i.e. 6.25% of the time.

```
# Curated movelist for ethane.

# Bond breaking: A-B -> A / B
move
natom 2
-
0 1
1 0
-
0 0
0 0
-
labels * *
prob 0.1

# Bond making: A / B -> A-B
move
natom 2
-
0 0
0 0
-
0 1
1 0
-
labels * *
prob 0.3

# H2 dissociation from a single carbon: H-C-H -> H-H / C
move
natom 3
-
0 0 1
0 0 1
1 1 0
-
0 1 0
1 0 0
0 0 0
-
labels H H C
prob 0.1

# H2 Association to a single carbon: H-H / C -> H-C-H
move
natom 3
-
0 1 0
1 0 0
0 0 0
-
0 0 1
0 0 1
```

```
1 1 0
-
labels H H C
prob 0.3

# A-C-B -> A-B / C
move
natom 3
-
0 0 1
0 0 1
1 1 0
-
0 1 0
1 0 0
0 0 0
-
labels * * *
prob 0.1

# A-B / C -> A-C-B
move
natom 3
-
0 1 0
1 0 0
0 0 0
-
0 0 1
0 0 1
1 1 0
-
labels * * *
prob 0.3

# A-C / B -> A-B / C
move
natom 3
-
0 0 1
0 0 0
1 0 0
-
0 1 0
1 0 0
0 0 0
-
labels * * *
prob 0.2

# A-B / C -> A-C / B
move
natom 3
-
0 1 0
1 0 0
0 0 0
-
0 0 1
0 0 0
1 0 0
-
labels * * *
prob 0.2
```

```
# A-B-C -> C-A-B
move
natom 3
-
0 1 0
1 0 1
0 1 0
-
0 1 1
1 0 0
1 0 0
-
labels * * *
prob 0.2
```

Figure S4: SE-GDS graph move library used in case study of 1000 K ethane pyrolysis.

S3.3 SE-GDS parameters

CDE input file for SE-GDS mechanism generation in direct CRN exploration of 1000 K ethane pyrolysis. Parameter definitions are documented in CDE's code repository at `https://github.com/HabershonLab/cde`.

Important parameters include:

- `startfile` - defines the geometry of the initial molecule system used for this SE-GDS run. This is usually generated by Kinetica when used within a CRN generation workflow.
- `valencerange` - defines acceptable valences for atom types. In this input, carbon atoms must be connected to 2-4 other atoms. Hydrogen atoms can be connected to 0 or 1 other atoms, allowing for hydrogen radical formation.
- `reactiveatomtypes` - defines atom types that are allowed to react.
- `nmcrxn` - number of mechanisms to generate, each of length `nrxn`.
- `nrxn` - number of reactions to sample per generated mechanism.

`nmcrxn` and `nrxn` are usually controlled by settings within Kinetica by the parameters `nrxn` and `radius` respectively within a CDE interface struct. When using the iterative exploration method, changing the latter two values creates CDE inputs with the former two values modified.

```
calctype breakdown
minmolcharge 0
maxmolcharge 0
nchargemol 0
maxstepcharge 0
maxtotalcharge 0
optaftermove .true.
ignoreinvalidgraphopt .false.
doinitialopt .false.
pesfull .false.
startfile Start.xyz

stripinactive .true.
optendsbefore .true.
optendsduring .false.

dofconstraints 0
atomconstraints 0

pestype xtb
```

```
pesfile xtb.head
pesopttype xtb
pesoptfile xtb.head
pesexecutable xtb --iterations 1000 --grad
pesoptexecutable xtb --input xtb.inp --iterations 1000 --etemp 1000 --opt tight --grad

movefile moves_2+3.in
gdsthresh 0.5
gdsspring 0.05
gdsrestspring 0.05
nbstrength 0.04
nbrange 2.5
kradius 0.05
ngdsrelax 10000
gdsdtrelax 0.1
gdsoutfreq 10
graphfunctype 4

valencerange{
C 2 4
H 0 1
}

reactiveatomtypes{
C
H
}

reactiveatoms{
all
}

reactivevalence{
}

fixedbonds{
}

allowedbonds{
}

nmcrxn 1
nrxn 100
```

Figure S5: SE-GDS parameters used in case study of 1000 K ethane pyrolysis.

S3.4 `RxFilter` Implementation

The `RxFilter` is a data-type within `KineticaCore` that can be passed into a CRN solution call along with the current CRN and a set of simulation parameters. The `RxFilter` allows for definition of an array of functions, each of which takes the current CRN (`sd` and `rd` representing instances of `SpeciesData` and `RxData` respectively) as its arguments and returns a mask of reactions to keep or remove. When a kinetic simulation is requested, each function's mask is calculated and accumulated into a global mask which removes the filtered reactions from the CRN. The filter used for removing reactions creating species with greater than four carbon atoms is shown in Figure S6.

```
function large_filter(sd, rd)
    mask = [false for _ in 1:rd.nr]
    for (i, prod) in enumerate(rd.prods)
        for pspec in prod
            if count(j->(j=='C'), pspec) > 4
                mask[i] = true
                break
            end
        end
    end
    return mask
end
filter = RxFilter([large_filter])
```

Figure S6: Creation of an `RxFilter` for removing reactions which create species with greater than 4 carbon atoms.
